# A fine-tuned vision transformer based enhanced multi-class brain tumor classification using MRI scan imagery

**DOI:** 10.3389/fonc.2024.1400341

**Published:** 2024-07-18

**Authors:** C. Kishor Kumar Reddy, Pulakurthi Anaghaa Reddy, Himaja Janapati, Basem Assiri, Mohammed Shuaib, Shadab Alam, Abdullah Sheneamer

**Affiliations:** ^1^ Department of Computer Science and Engineering, Stanley College of Engineering and Technology for Women, Hyderabad, India; ^2^ Department of Computer Science, College of Engineering and Computer Science, Jazan University, Jazan, Saudi Arabia

**Keywords:** MRI scans, deep learning models, vision transformers, FTVT, medical image processing

## Abstract

Brain tumors occur due to the expansion of abnormal cell tissues and can be malignant (cancerous) or benign (not cancerous). Numerous factors such as the position, size, and progression rate are considered while detecting and diagnosing brain tumors. Detecting brain tumors in their initial phases is vital for diagnosis where MRI (magnetic resonance imaging) scans play an important role. Over the years, deep learning models have been extensively used for medical image processing. The current study primarily investigates the novel Fine-Tuned Vision Transformer models (FTVTs)—FTVT-b16, FTVT-b32, FTVT-l16, FTVT-l32—for brain tumor classification, while also comparing them with other established deep learning models such as ResNet50, MobileNet-V2, and EfficientNet - B0. A dataset with 7,023 images (MRI scans) categorized into four different classes, namely, glioma, meningioma, pituitary, and no tumor are used for classification. Further, the study presents a comparative analysis of these models including their accuracies and other evaluation metrics including recall, precision, and F1-score across each class. The deep learning models ResNet-50, EfficientNet-B0, and MobileNet-V2 obtained an accuracy of 96.5%, 95.1%, and 94.9%, respectively. Among all the FTVT models, FTVT-l16 model achieved a remarkable accuracy of 98.70% whereas other FTVT models FTVT-b16, FTVT-b32, and FTVT-132 achieved an accuracy of 98.09%, 96.87%, 98.62%, respectively, hence proving the efficacy and robustness of FTVT’s in medical image processing.

## 1 Introduction

**Figure 1:**
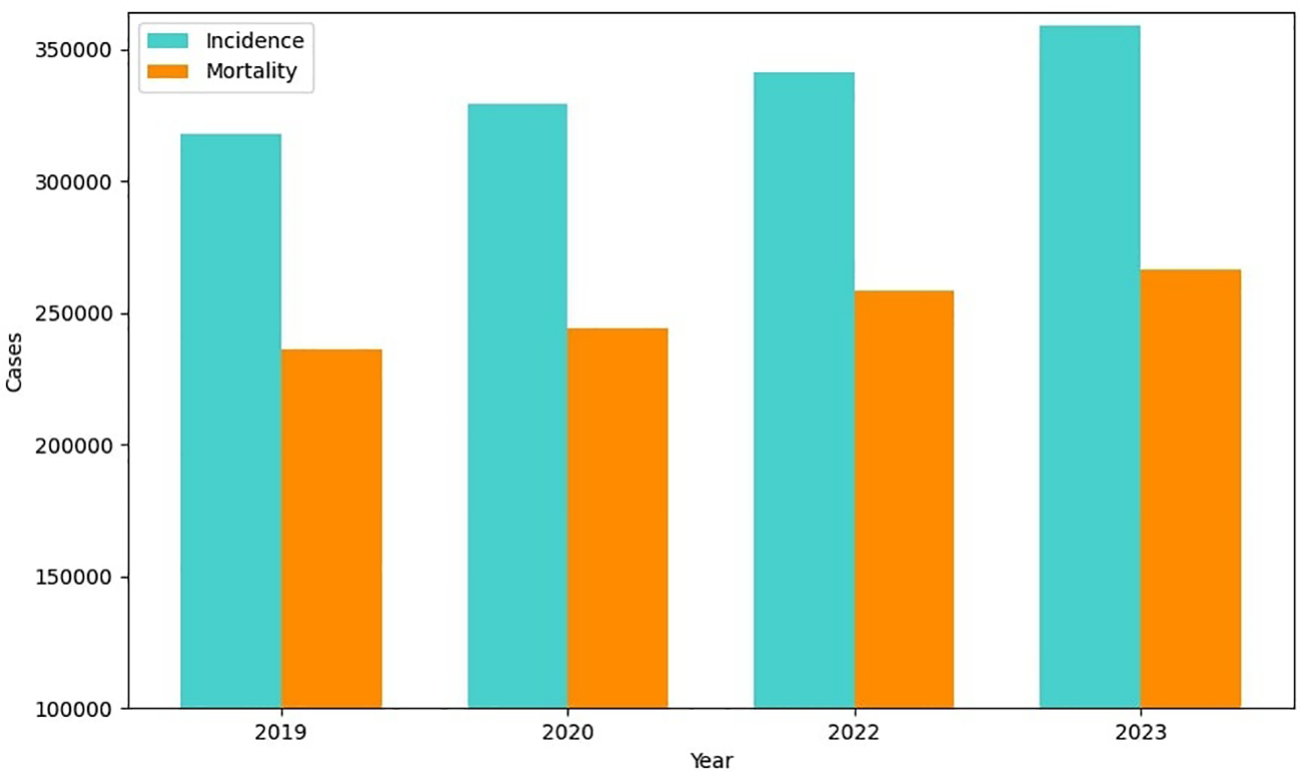
Brain tumor incidence and mortality worldwide.

A tumor in the brain is an abnormal mass or cell that grows inside the brain or central nervous system. The indicators of a brain tumor depend on its size, position, and rate of progression. Symptoms include headaches, seizures, and cognitive deficits (1). Diagnostic techniques involve MRI scans or CT scans followed by biopsy for further identification of the disease. Surgery, targeted therapy, radiation therapy, chemotherapy, or combination of any of these methods can be used as treatment options (2). Tumors encompass both cancerous (malignant) and non-cancerous (benign) growths, with cancers specifically referring to malignant tumors. Nevertheless, not every tumor is cancerous since all cancers grow in the form of tumors (1). Compared to other types of cancer, brain tumors have a shorter survival rate. Early diagnosis of brain tumors presents a challenging task because of its irregularity, morphology, heterogeneous location, and blurred boundaries. Accurate tumor characterization at this stage enables clinicians to make better treatment decisions. There are two types of tumor: primary and secondary. In primary tumor, the tumor cells develop in the brain first before spreading to other parts of the body, and in secondary or metastatic tumor, the tumor cells start in other parts of the body and then spread to the brain (3). The proposed study aims to classify glioma, meningioma, and pituitary brain tumor. This study particularly focuses on classification on these types of tumors due to their distinct diagnostic challenges and differences in location, behavior, and method of treatment. They are clinically significant tumors which require accurate classification which in turn requires sufficient data for training and testing the Deep Learning (DL) models effectively. Glioma develops from glial cells that support neurons in the brain; these are primary tumors. In adults, the meningioma is commonly seen, and subtypes include astrocytoma, oligodendroglia, and ependymoma (4). Meningioma arises from the protective nerves of the brain and tends to be benign and grow slowly. These are more common in women and the older adults and both may have different symptoms and need different treatments (5). Pituitary tumors, from the pituitary gland, can be benign or malignant, with most being benign and they grow slowly (6). The data in **Figure 1** show for brain tumor incidence and mortality have increased sequentially for 4 years.. Although there was a slight increase in reported cases in 2020 compared to 2019 (7), there was a slight increase in mortality and the following years have seen notable rise in reported cases and mortality. This change indicates the increase of brain tumors, which suggests that this topic deserves attention and further research. Overall, the information presented in **Figure 1** highlights the importance of ongoing research and interventions aimed at providing accurate results.

Machine learning to DL techniques are employed in brain tumor classification due to their ability to extract intricate patterns and features from MRI images efficiently. These advanced algorithms enhance the precision and efficiency of tumor diagnosis. In brain tumor classification, the machine learning algorithms analyze MRI images to identify patterns such as texture and longitudinal patterns; features such as intensity variations, shape, and size; and statistical features such as mean intensity to classify. The machine learning techniques used include K-Nearest Neighbors (KNN) (8), an instance-based learning; ensemble learning methods like ExtraTrees (ET) (8); Random Forest (9); a combination of ensemble learning methods such as Ada and Random Forest (10); a combination of ensemble learning as classifier with feature extractors like Random Forest (RF) classifier and for feature extraction using TGA (Tree Growth algorithm); LBP (Local Binary Pattern) and GLCM (Gray level co-occurrence matrix) (11); and a probabilistic technique, namely, Naive Bayes (8), which are also used in brain tumor classification. They are effective techniques but come with fair share of drawback. Random Forest is effective for high-dimensional data but can over fit noisy data. KNN’s performance may degrade with high-dimensional data and requires a suitable distance metric for image data. SVM’s effectiveness heavily relies on choosing the right kernel and appropriate regularization parameters, which can be challenging. XGBoost performance may deteriorate with noisy data and could be computationally expensive. ET might struggle with small and noisy datasets. Naive Bayes assumes independence among features, which might not hold true for image data, potentially impacting its performance.

CNNs (convolutional neural networks) and DL techniques have attested to their ability to automatically extract relevant features from un-processed MRI image data, thus providing improved accuracy for brain tumor classification (12). CNN models especially pre-trained models, that is, trained on large dataset (13) leverage transfer learning, models such as Inception V3, VGG 16, and Resnet-50 (14) have shown exceptional performance in classifying brain tumors on different datasets as they can learn complex representations. Auto-encoders in combination with DL models like ResNet (15) and machine learning models like KNN (16) were used in recent studies. However, to avoid overfitting and to generalize well, huge loads of data for training are required by CNNs (17), computationally intensive and require high processing power, especially for large-scale applications; they struggle with translation equivariance, that is, highly responsive to small changes in input data, such as changes in lighting and orientation, which can affect their performance.

ViTs (Vision Transformers) can effectively process long-range dependencies in data, overcoming the limitations of machine learning and CNNs in handling global information (18). ViTs are highly parallelizable, making them suitable for scalable and efficient training on modern hardware. They offer improved interpretability through feature learning mechanisms called the multi-head self-attention modules, allowing for a better understanding of model predictions (18). ViTs can handle objects at different scales within the same image effectively due to their self-attention mechanism, reducing the need for handcrafted scale-specific features. These models are more robust to changes in the input data, because they can understand how different parts of the input are connected, with the help of a special technique called self-attention (18). The need for extensive medical imaging data and reducing computational resources, potential overfitting limits the novelty of the ViT models which must be addressed. To reach their full potential, ViTs require a substantial amount of training data. However, collecting such extensive data is a challenge in medical imaging. To address this limitation, recent efforts, as described in (19), have resulted in several pre-trained and optimized models trained on large datasets, ImageNet21k and ImageNet2012. Notably, these approaches have demonstrated success in various medical imaging diagnostics (20, 21). The proposed study introduces Fine-Tuned Vision Transform models (FTVTs) with updated classifier heads, tuned the hyper-parameters for efficient brain tumor classification and along the same lines developing the DL models and comparing their performance.

The following discussion explores the studies of eight authors, investigating the various datasets, algorithms, and methods they have employed. The initial focus is on ViTs in the research of the first two authors, while the remaining studies are centered around a range of DL models.

In his work using the dataset consisting 3064-MRI scans of the brain from figshare, the author Sudhakar Tummala et al., (22) developed ViTs and gained an approximate accuracy of 98% for all the models. Various optimizer and hyperparameter values for each optimizer were tested. For the classification of brain tumor, an ensemble of the model’s results were used. In the proposed study, both the DL and FTVT models feature less complex architectures. These models have updated classifier heads using Dropout, linear, and Batch Normalization (BN) layers, meticulously tailored for brain tumor classification, a feature not observed in the previous study. Additionally, fine-tuning in the proposed study led to significantly higher values for accuracy, F1 score, precision, and recall.

Abdullah A. Ansari et al. (23) used a database of 5,712 MRI images and performed classification of brain tumors using a neural network model, with the pre-trained ViTs as the initial layer and introduced BN, Dense layers for task-specification, and developed five models R50-ViT-l16, ViT-l16, ViT-l32, ViT-b16, and ViT-b32. With complex architecture, high accuracy values are gained.

Mallika.M Badža et al. (24) presented a custom CNN model for brain tumor classification from the images collected from various Chinese hospitals from the years 2005–2010 and recorded results using 10-fold cross-validation methods and different datasets containing 3,064 MRI images and obtained an accuracy of 96%. The architecture of this model is simple and fails to study complex patterns.

A pairwise GAN (generative adversarial network) model is used to classify various types of glioma brain tumors by Chenjie Ge et al. (25) and gained a mean accuracy of 88.82% and augmented MRI images are added to overcome the insufficiency of images for classification.

Zhiguan Huang et al. (26) used a dataset named “Segmentation Labels for the Pre-operative Scans of the TCGA-LGG collection” and proposed a complex CNN model in which randomly generated graph algorithms are used for building the network with an activation function modification obtained an accuracy of 95.49%. The results obtained are visually represented using scatterplots and compared with various CNN models such as ResNet, DenseNet, MobileNet, and EfficientNet models, which acquired lower accuracies in comparison with the suggested model.

Gokalp Cinarer et al. (27) studied sophisticated DNN (deep neural network) model in combination with DWT (discrete wavelet transform) for glioma subtype classification where the brain tumor MRI scans were collected from (TCIA) Cancer Imaging Archive portal, the model classifies the MRI scans into four grades as per the World Health Organization and with appropriate feature selection and extraction techniques the model recorded an accuracy of 96.15%.

Ayadi Wadhah, et al. (28) proposed a classification model using feature extractors D-SURF (Descriptor Speeded Up Robust Features) and HoG (Histogram of Oriented Gradients). For the classification process, an SVM (Support Vector Machine) is used for a dataset of 3,064 images in their work. Even with the careful process for feature extraction, an accuracy of 90.27% is achieved which suggests that further fine-tuning is required for SVM.

A comparative analysis of models CNN, Inception-V3, VGG 16, and Resnet-50 on a dataset containing 3,264 MRI images was performed by Md Ishtyaq Mahmud et al, (14) achieved accuracy in the range of 80%–93.30%, respectively. For data preprocessing, Gaussian and Laplacian filters were employed. A complex CNN architecture, pretrained ResNet-50, Inception-V3, and VGG-16 models are used for brain tumor classification. A detailed overview for the models was provided.

**Table 1** contains all the brief descriptions of the studies mentioned in relevant.

**Table 1:** List of all the authors and the dataset in the relevant work.

SNo.	Author name	Dataset used	Proposed method	Disadvantages
1.	Abdullah A. Asiri, et al. (23)	5,712 MRI images	ViT-b16,b32, l16, l32, R50-ViT-l16,	1. Complex architecture 2. Low accuracy values observed for individual brain tumor classes.
2.	Sudhakar Tummala, et al. (22)	TCIA (29, 30)	ViT B16,B32,L16, L32	1. Measures for preventing feature overlapping between tumor classes meningiomas and gliomas, meningioma, and pituitary are not taken.
3.	Mallika Naser et al. (24)	Figshare (31)	CNN Model	1. The augmented dataset’s accuracy was only 88%. 2. Classification error rates are higher compared to other CNN models.
4.	Chenjie Ge et al. (25)	Cancer Imaging Archive (TCIA) (32)	Pairwise GAN	1. Accuracy of the model depends on size of dataset used, worked well for small datasets. 2. Only glioma subtype classification is performed.
5.	Zhiguan Huang et al., (26)	Figshare (31)	CNN models (ResNet, DenseNet, EfficientNet, MobileNet)	1. Complex architecture generated through graph algorithms and network generators. 2. Higher training time for the models (in minutes).
6.	Gokalp Cinarer et al. (27)	TCIA (33)	DNN with DWT (discrete wavelet transform)	1. Model is trained only to detect and classify various glioma brain tumor grades. 2. The size of the dataset is very less with only 121 images of brain tumor scans.
7.	Wadhah Ayadil et al. (28)	3064 MRI images	DSURF and HoG with SVM	1. SVM with a linear kernel is used for classification, which achieved 90.27% accuracy which suggests absence of fine tuning.
8.	Md Ishtyaq Mahmud et al. (14)	3264 MRI images	Inception V3,VGG 16 and Resnet-50	1. Pre-trained models lacked fine tuning as result low accuracies ranging from 80% to 93.3%, which are significantly low for deep learning models. 2. Complex CNN architecture was employed

## 2 Materials and methods

### 2.1 Dataset used

**Figure 2:**
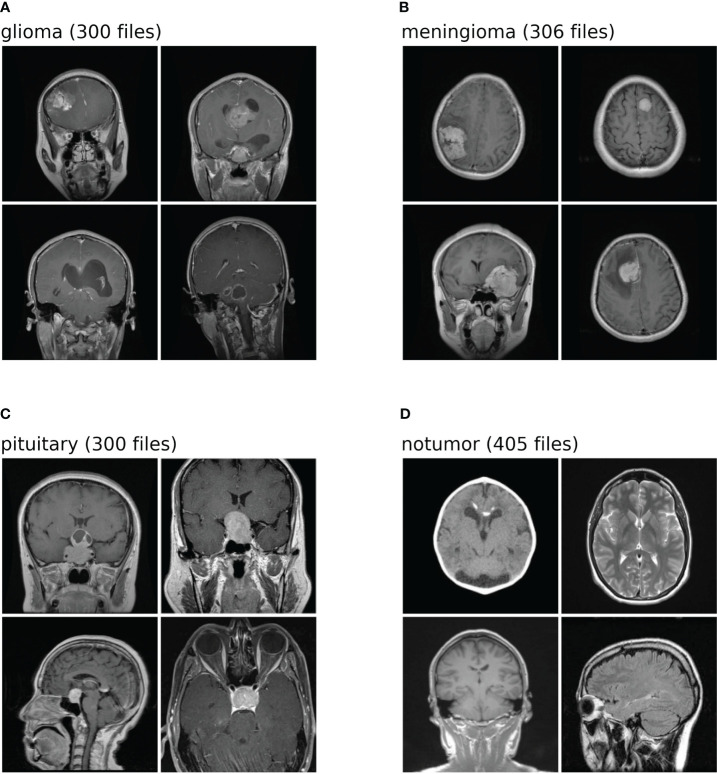
Shows the dataset comprises images of four distinct classes. Each class is identified by a specific label: **(A)** denotes glioma; **(B)** corresponds to meningioma; **(C)** represents pituitary; **(D)** signifies no tumor. The numeric values associated with each class label is the number of test images within the dataset for brain tumor classification task.

The dataset used in this analysis is a combination of three-distinct datasets, namely, figshare, SARTAJ, and Br35H datasets. The figshare dataset on brain tumors comprises 3,064 brain scan images of three classes of brain tumors captured via T1-weighted contrast-enhanced imaging. The SARTAJ dataset is a directory of folders containing MRI data in which the images are already split into Training and Testing folders, which again are divided into four subfolders. These subfolders encompass MRI scans of corresponding tumor classes. The third dataset Br35H contains three folders namely yes, no, and pred. The yes and no folders include 1,500 MRI scans that are classified as tumorous and non-tumorous, respectively. Altogether, the dataset has 7,023 images of MRI scans that are classified into four distinct classes, namely, glioma, meningioma, pituitary, and no tumor as shown in the **Figure 2**. In this research, 5,712 images are utilized for training and 1,311 other images for testing. A glimpse of the dataset is shown in the **Figure 2**.

**Figure 3:**
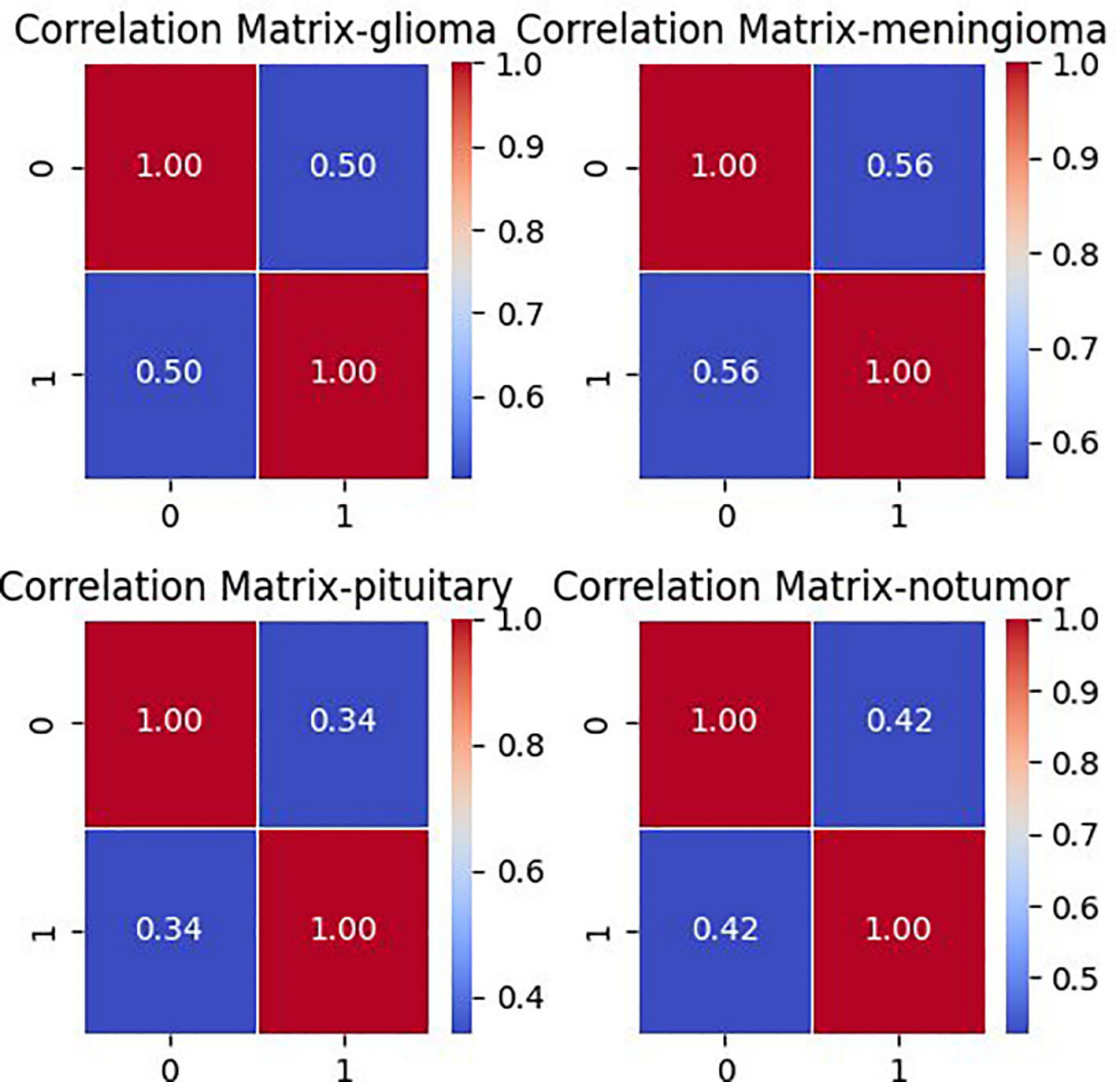
Correlation matrix for each class in brain tumor MRI dataset.

In the brain tumor classification dataset, a correlation matrix is calculated using Pearson correlation coefficient. This matrix depicts the correlation between pixel values across all pairs of images within each class. Samples of 100 images per class are chosen, and pairs of images are examined. For each pair, the Pearson correlation coefficient is computed, signifying the degree and direction of their linear relationship. Subsequently, an average correlation matrix is derived from these coefficients. **Figure 3** shows this average correlation matrix. Each cell in the matrix reflects the average correlation coefficient between the pixel values of two images, offering insight into the overall similarity or dissimilarity of pixel values in the dataset.

### 2.2 Data preprocessing on “brain tumor classification (mri)” dataset

Data preprocessing is a machine learning technique that can convert any input data into a suitable format that is more useful for training the model. This study implemented some image augmentation and transformation techniques such as image resizing, flipping, rotating, jittering, and normalization using transform.compose() function from torchvision library. Data augmentation is an essential technique in machine learning, particularly for tasks such as image classification. By applying transformations such as resizing, flipping, rotating, and jittering to the input data, augmentation increases the robustness of the model by exposing it to variations in the data, allowing it to recognize patterns in different orientations, sizes, and positions. This, in turn, leads to improved generalization, as the model becomes less likely to overfit to the training dataset and can better handle variations in real-world data. Augmentation enhances the performance of the model, particularly on unseen data, by diversifying the training dataset and enabling the model to learn from a wider variety of examples. It also serves as a form of regularization, reducing overfitting by introducing noise and variations into the training data. Overall, data augmentation plays a critical role in enhancing the performance, robustness, and generalization ability of machine learning models. All the input scans are resized to 224 × 224 sized pixels; the images are then horizontally flipped by an angle of 10° with a 0.5 probability and rotated. The “0.5 probability” refers to the chance or likelihood of the horizontal flipping being applied to each image. It indicates a 50% probability, meaning there’s a 50/50 chance for each image to undergo horizontal flipping. This randomness helps introduce diversity into the dataset, enhancing its quality and allowing the model to learn from various perspectives. To adjust the brightness, contrast, hue, and saturation, a data augmentation technique called color jitter is applied. The images are then transformed into PyTorch tensor format from PIL image format. In the next step, image normalization is performed to normalize the pixel values of images, which improves training and performance of the model. The images are then split into three sets training, testing, and validation—with random_split() function. Applying preprocessing techniques such as transformation and augmentation increases the quality, volume, and diversity of image data. It also helps in model regularization, prevent overfitting, and to obtain better model performance.

### 2.3 Brain tumor classification methods

This section comprehensively examines two prominent methodologies employed for brain tumor classification: DL models and FTVT. The discussion commences by providing a detailed explanation of the architectural design principles underlying each model. Subsequently, the implementation of these models for the specific task of brain tumor classification is meticulously explored. Finally, a rigorous comparison is undertaken to evaluate the overall performance of both DL models and their FTVT counterparts.

### 2.4 Deep learning models for multi-class brain tumor classification using MRI scan imagery

**Figure 4:**
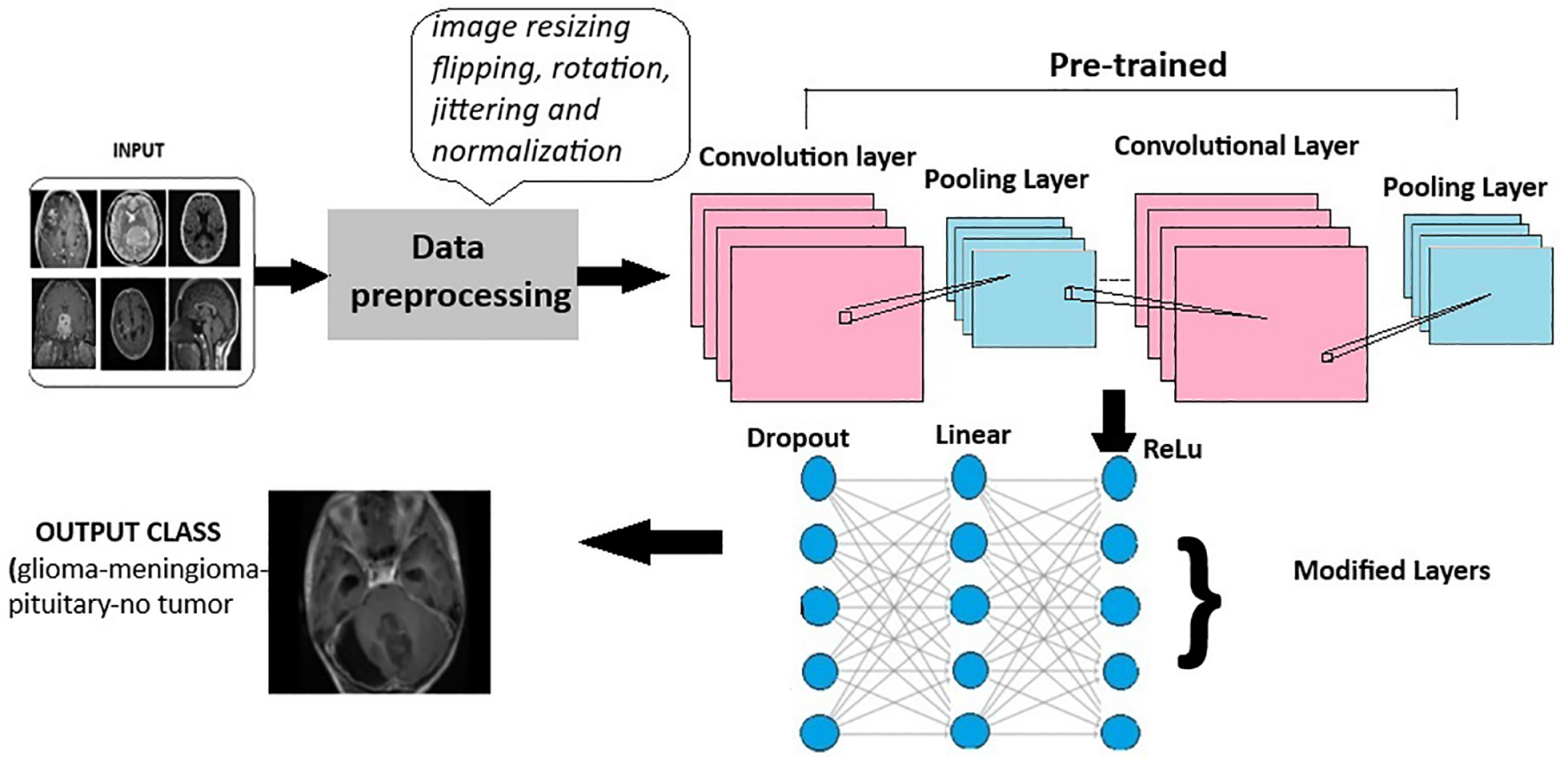
General architecture for the deep learning models for multi-class brain tumor classification using MRI scan imagery.

Deep learning models leverage advanced neural network architectures to automate the process of identification and classification of brain tumors for magnetic resonance imaging (MRI) scans. These architectures may be CNNs (Convolution Neural Networks), FNNs (Feedforward Neural Networks), RNNs (Recurrent Neural Networks), or more recent FTVTs. Trained on a large number of datasets containing labeled MRI images, the model can detect tumor formations on any newly presented MRI image with great accuracy. Let us first understand the general architecture of deep learning models **Figure 4**.

#### 2.4.1 Architecture of deep learning models

In deep learning models, a brain MRI scan acts as primary input for the model. The MRI scan produces high-resolution, three-dimensional images of the brain, where varying shades of gray represent different tissue characteristics, thus helping in tumor detection. Then, input image is resized if necessary, apart from image resizing various preprocessing techniques are applied including image jittering and normalization. The pretrained model is initiated, which contains convolution and pooling layers where convolution layers filter the input and extract features, dimensionality reduction is performed by the pooling layer.

A combination of Dropout, Linear, and ReLU layers is added to the model for prevention of overfitting and better model performance. The output is a classification of the input image into the appropriate brain tumor class. The model classifies the input brain MRI scan into corresponding brain tumor types (glioma-meningioma-pituitary or no tumor). ReLU6 is an activation function that is generally the modification of ReLU, that is, Rectified Linear Unit. The ReLU6 and ReLU activation function can be mathematically defined as


ReLU6 f(x)=min(max(x,0), 6) 

ReLU  f(x)=max(x,0)

ReLU6 limits the activation to a size of 6, which increases the functions robustness along with decreasing the precision computations.

**Figure 5:**
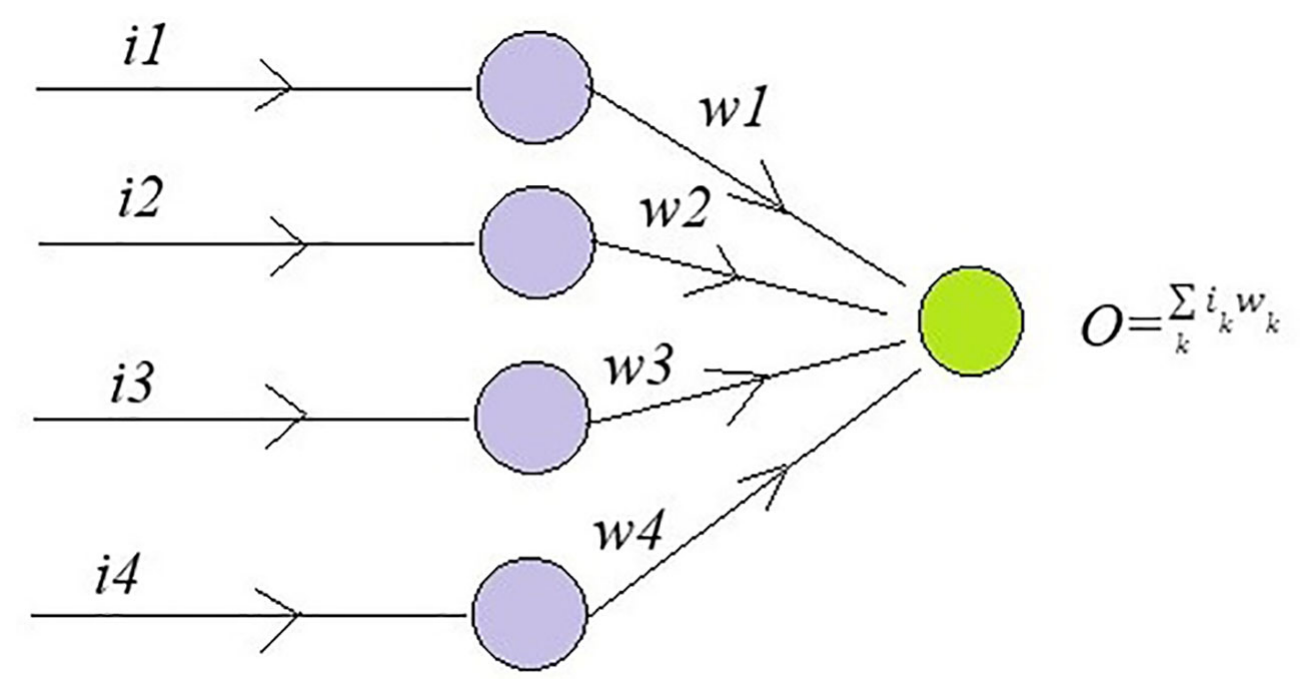
A single-linear input layer of neural network to understand the Dropout layer.

To understand Dropout, consider a single-linear input layer of neural network (**Figure 4**) for simplicity with inputs as *i_k_* and corresponding weights as *w_k_* where *k* = 1–4, the below **Figure 5** helps in understanding the working of Dropout layer.

The output is the sum of product of inputs and weights. The error rate is calculated as


$$E_{D}=1/2\left(t-\sum_{k=1}^{n}{\text{δ}}_{i}w_{k}i_{k}\right)$$

where *t* is target value and δ is a drop rate which lies between 0 and 1.The error is back propagated to find gradient.


$$\frac{\text{δE}}{{\text{δw}}_{\text{k}}}=\;-{\text{tδ}}_{\text{k}}{\text{i}}_{\text{k}}+{\text{w}}_{\text{k}}{\text{δ}}_{\text{k}}^{2}{\text{i}}_{\text{k}}^{2}+\sum_{\text{j}\neq 1\neq \text{k}}^{n}{\text{w}}_{\text{j}}{\text{δ}}_{\text{k}}{\text{δ}}_{\text{j}}{\text{i}}_{\text{k}}{\text{i}}_{\text{j}}$$

This equation calculates the expectation of concerning the gradient of the normal error rate $E_{N}$ where *p* is Bernoulli (*p* = 0 or 1) and it is gradient $\frac{\text{δ}E}{\text{δ}w_{k}}$ , we get the below equation.


$$E_{N}=1/2(t-\sum_{k=1}^{n}p_{k}w_{k}i_{k}{)}^{2},$$

$$\frac{\text{δ}E}{\text{δ}w_{k}}=\;-t{\text{δ}}_{k}i_{k}+w_{k}{\text{δ}}_{k}^{2}i_{k}^{2}+\sum_{j\neq 1\neq k}^{n}w_{j}{\text{δ}}_{k}{\text{δ}}_{j}i_{k}i_{j}$$

$$\text{E}\left[\frac{\text{δE}}{{\text{δw}}_{\text{k}}}\right]=\;\frac{{\text{δE}}_{\text{N}}}{{\text{δw}}_{\text{k}}}+{\text{w}}_{\text{k}}{\text{p}}_{\text{k}}\left(1-{\text{p}}_{\text{i}}\right){\text{i}}_{\text{k}}^{2}$$

Equation 4 is equal to the gradient of Eɴ if *w* = p*w. To obtain maximum regularization *p* = 0.5.

### 2.5 Deep learning models

#### 2.5.1 ResNet-50

**Figure 6:**
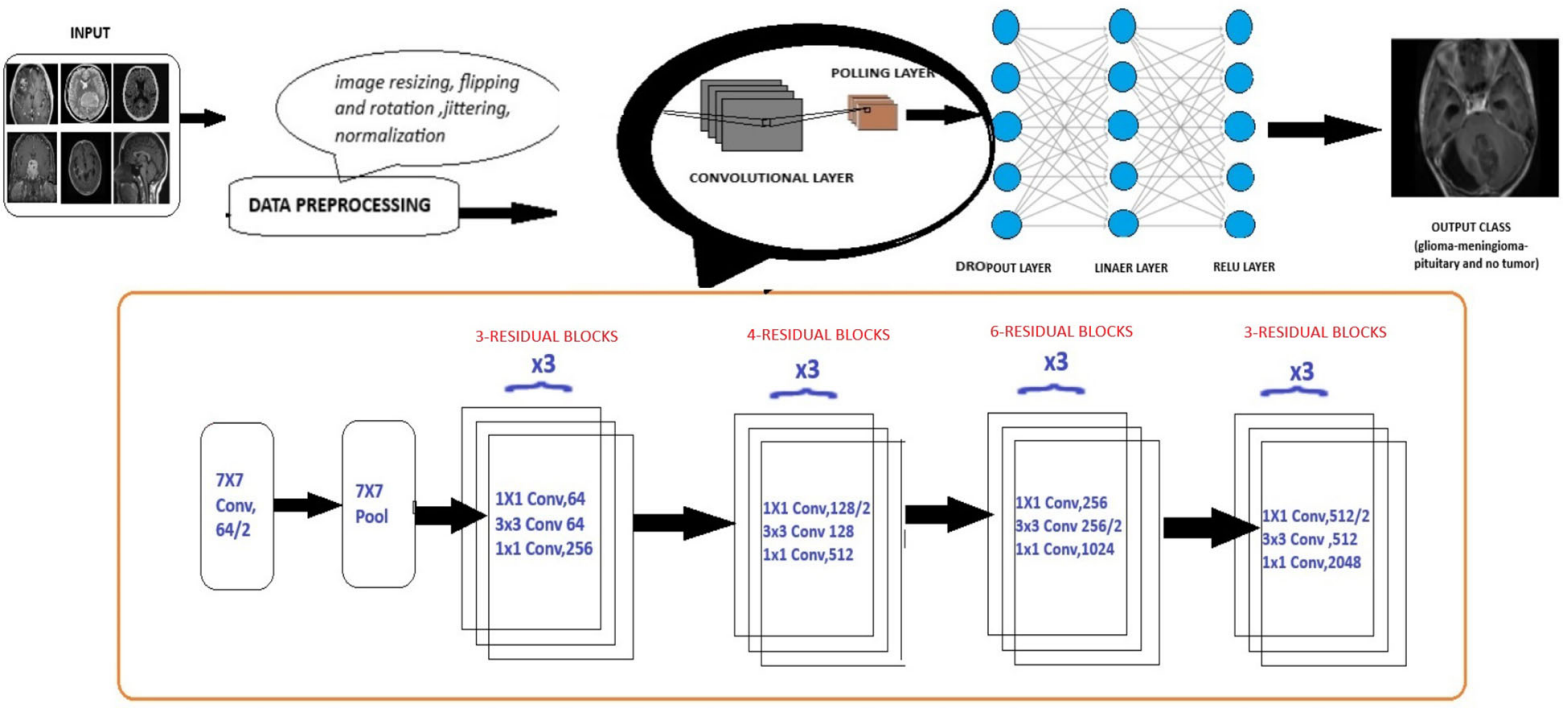
Extension of general architecture to ResNet-50 for brain tumor classification task.

ResNet (Residual Network) model is a CNN consisting 50 layers (48 convolutional layers, a max pool layer, and an average pool layer), which is a pretrained network trained on over a million images and can classify them into 1,000 object categories. The model is applied on the brain tumor dataset (224 × 224—input image size) with required adjustments to detect brain tumor and its types. Dropout, Linear, and ReLU layers are added for better computation and to prevent overfitting. The final fully connected layer is altered to enable multi-class classification. During fine-tuning, various parameters of ResNet-50 are adjusted with respect to complexities identified. Stochastic Gradient Descent (SGD) optimizer optimizes the model’s parameters with a learning rate = 0.001, momentum = 0.7, and weight decay = 1e−4. In the training process, a scheduler is introduced for learning rate modification to gain better accuracy. The model’s performance is calculated after each epoch to analyze the overall model’s performance. The detailed architecture of ResNet-50 is depicted in **Figure 6**. Like any other convolutional network, the architecture of ResNet-50 starts with a convolution layer that performs filtering and feature extraction with a pooling layer following it. The pooling layer performs dimensionality reduction, thus reducing the model’s complexity. After the pooling layer, there are 48 layers—a division of layers into four residual blocks (9 + 12 + 18 + 9). The ResNets help the model to learn complex patterns. ResNet-50 includes concepts such as residual learning and bottle-neck blocks.

Consider an initial mapping *I(x)* where “*x*” is input, instead of approximating this mapping function directly to output function, residual learning re-parameterized the function to

**Figure 7:**
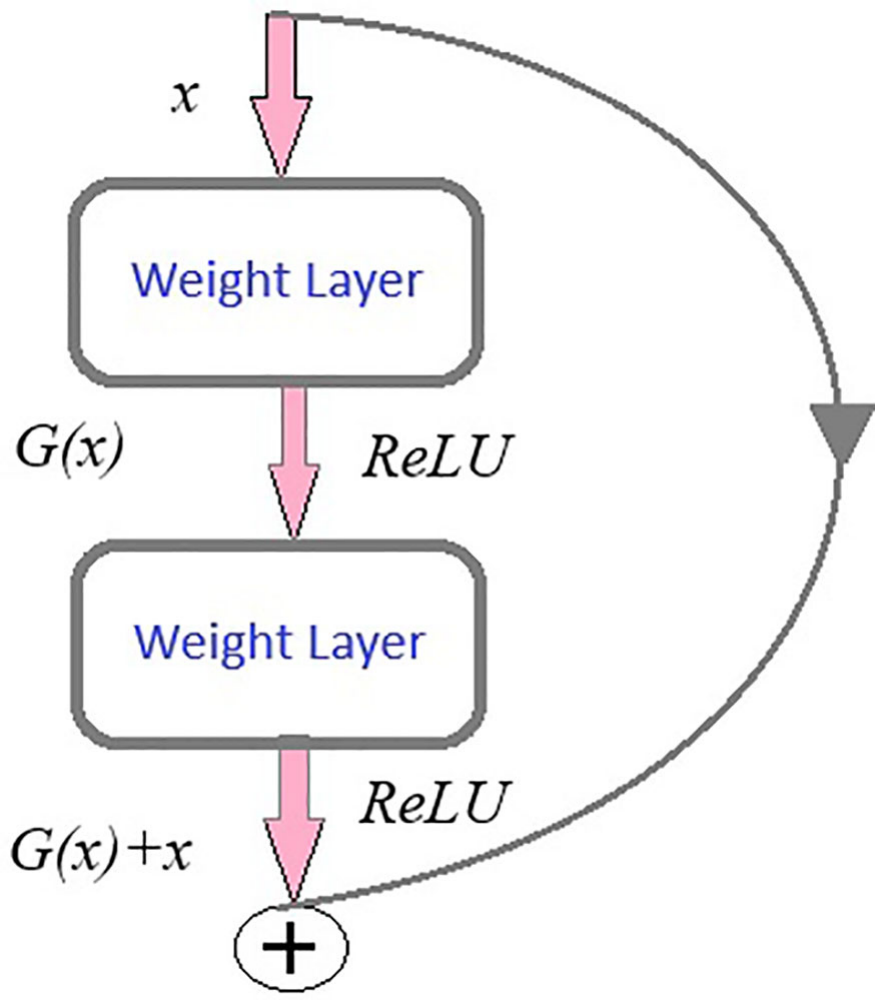
Residual learning.

*G(x) = I(x)-x* thus representing output as z*=G(x) + x* as shown in **Figure 7**. ResNet is based on skip connections. It includes skipping of layers which helps in regularization.

Another key concept of ResNet includes bottleneck blocks. This block is a sequence of 3- convolutional network layers. The initial one is a 1 × 1-convolution layer whose role is dimensionality reduction, followed by a 3 × 3 convolution layer which is responsible for extraction of features and the last layer is again an 1 × 1 convolution layer with its function-to restore dimensions. These layers are shown in **Figure 4**.

#### 2.5.2 EfficientNet-B0

EfficientNet model is a CNN similar to the first model with an additional concept called compound scaling [29]. It is a pretrained network trained on over a million images and can classify them into 1,000 object categories. The model is applied on the brain tumor dataset (224 × 224—input image size) with required adjustments to detect brain tumor and its types. Dropout, Linear, and ReLU layers are added for better computation and to prevent overfitting. The final fully connected layer is altered to enable multi-class classification. During fine-tuning, various parameters of efficientnet_b0 are adjusted with respect to complexities identified. SGD optimizer optimizes the model’s parameters with a learning rate = 0.001, momentum = 0.7, and weight decay = 1e−4. In the training process, a scheduler is introduced for learning rate modification to gain better accuracy. The model’s performance is calculated after each epoch to analyze the overall model’s performance.

**Figure 8:**
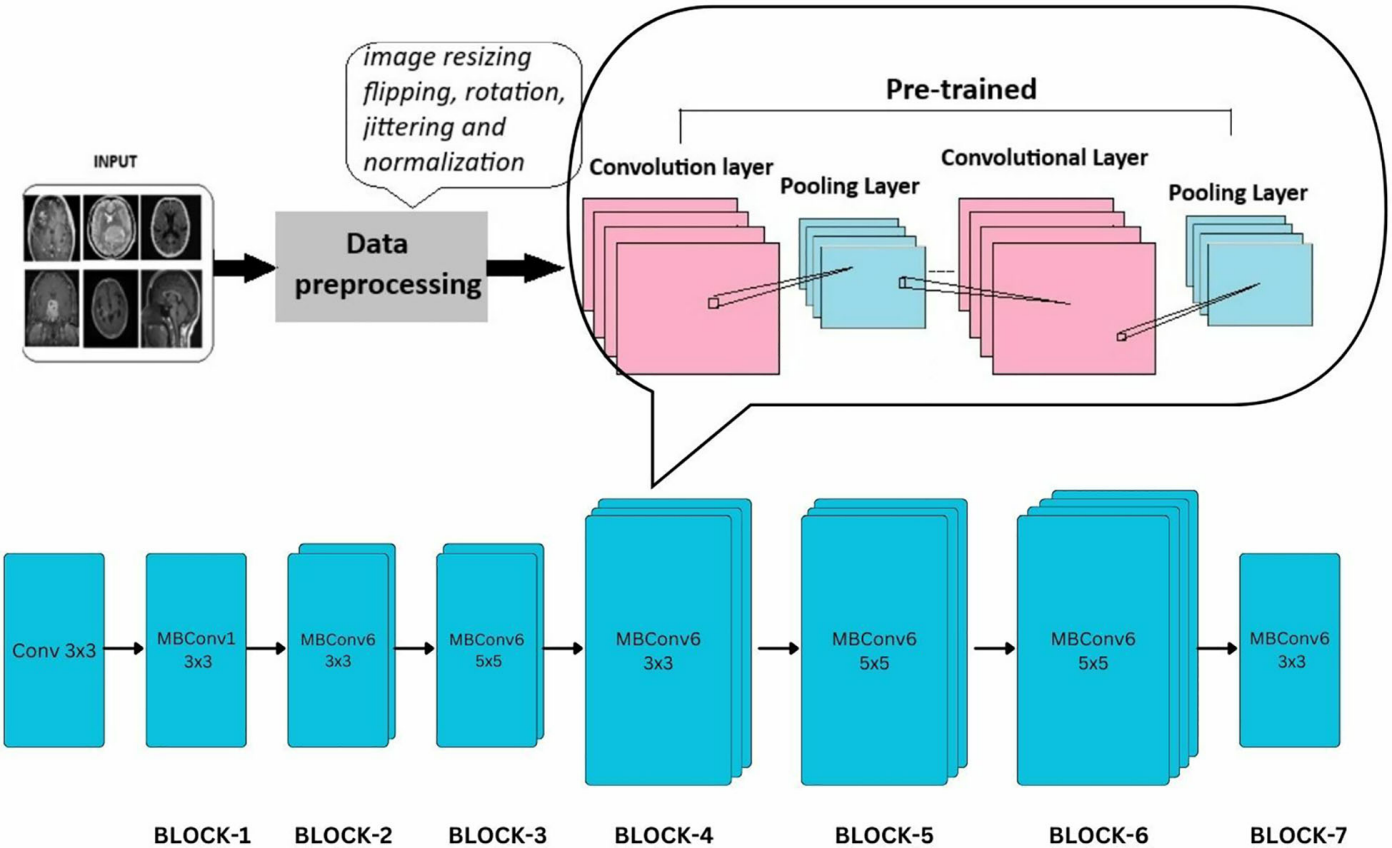
Extension of general architecture to EfficietNet-B0 for brain tumor classification task.

The EfficientNet-b0 model architecture contains a series of MBConv layers, which are nothing but mobile inverted bottleneck convolution layers. As its name implies, the MBConv layers are inverted versions of bottleneck blocks discussed in ResNet-50, which have the same three layers and functions but in reverse. The detailed layers are shown in **Figure 8**.

#### 2.5.3 MobileNet-V2

MobileNet model is a CNN developed for small-scale classifications. It is a lightweight neural network model designed for mobile services. The model was applied to the brain tumor dataset (224 × 224—input image size) with required adjustments to detect brain tumors and their types. Dropout, Linear, and ReLU layers are added for better computation and to prevent overfitting. The final fully connected layer is altered to enable multi-class classification.

**Figure 9:**
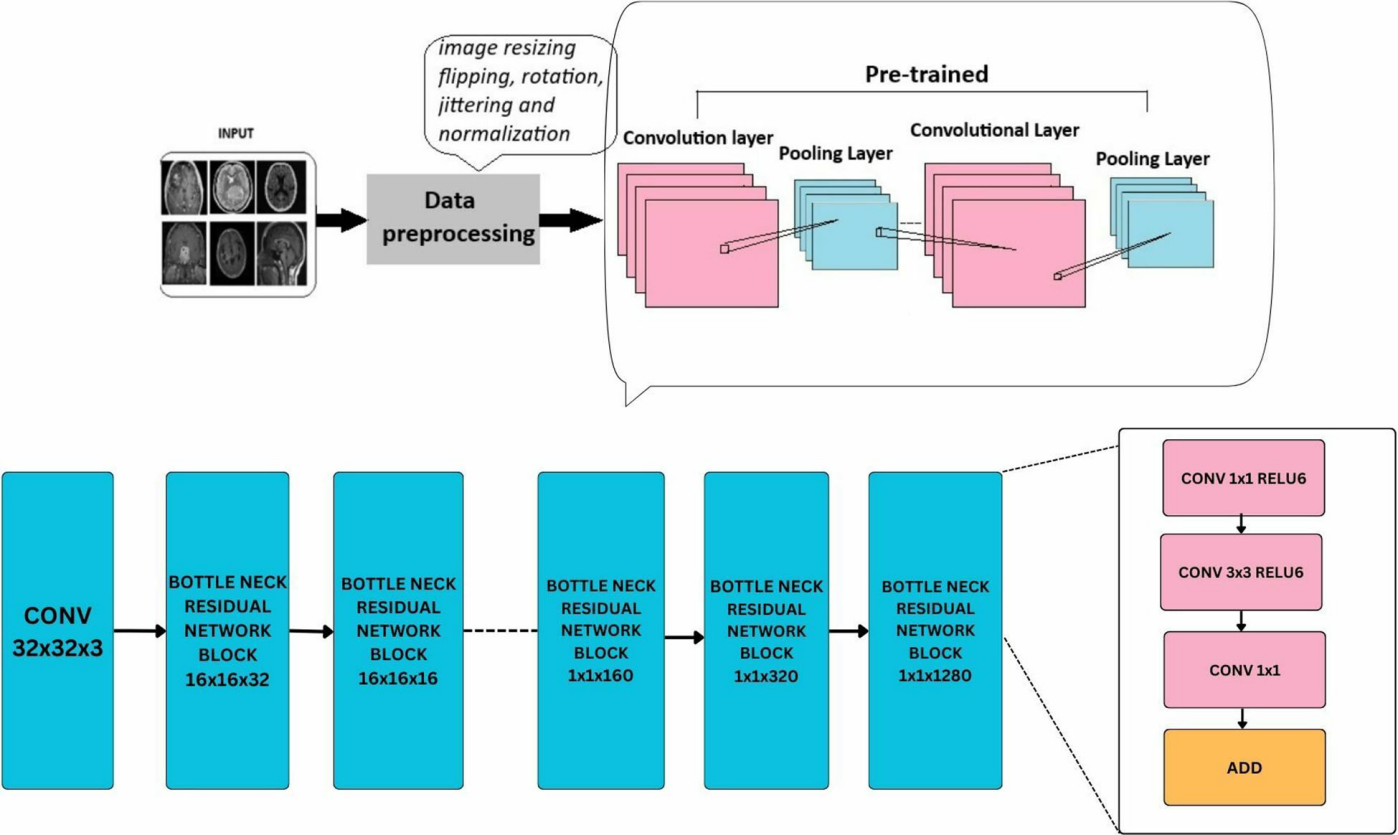
Extension of general architecture to MobileNet-V2 for brain tumor classification task.

During fine-tuning, various parameters of mobilenet_v2 are adjusted with respect to the complexities identified. SGD optimizer optimizes the model’s parameters with a learning rate = 0.001, momentum = 0.7, and weight decay = 1e−4. In the training process, a scheduler is introduced for learning rate modification to gain better accuracy. The model’s performance is calculated after each epoch to analyze the overall model’s performance. **Figure 9** shows a detailed architecture of MobileNet-v2; it includes a series of 17 bottleneck blocks with different sizes, which are similar to the one discussed above. The bottleneck blocks contain a 1 × 1 convolution layer that uses activation function ReLU6, a 3 × 3 convolution layer with the same activation, and the last is again a 1 × 1 convolutional layer without any activity function.

### 2.6 Vision transformers for multi-class brain tumor classification using MRI scan imagery

ViTs are progressive deep-learning models designed specifically for computer vision tasks. Following the impressive results of transformers in NLP (natural language processing), ViTs have revolutionized the field by introducing a novel approach to processing visual data. Unlike traditional CNNs, which perform on grid-like structures, ViTs deal with images as sequences of tokens, permitting them to efficiently capture worldwide information and complicated spatial relationships inside images.

#### 2.6.1 General architecture for vision transformers

**Figure 10:**
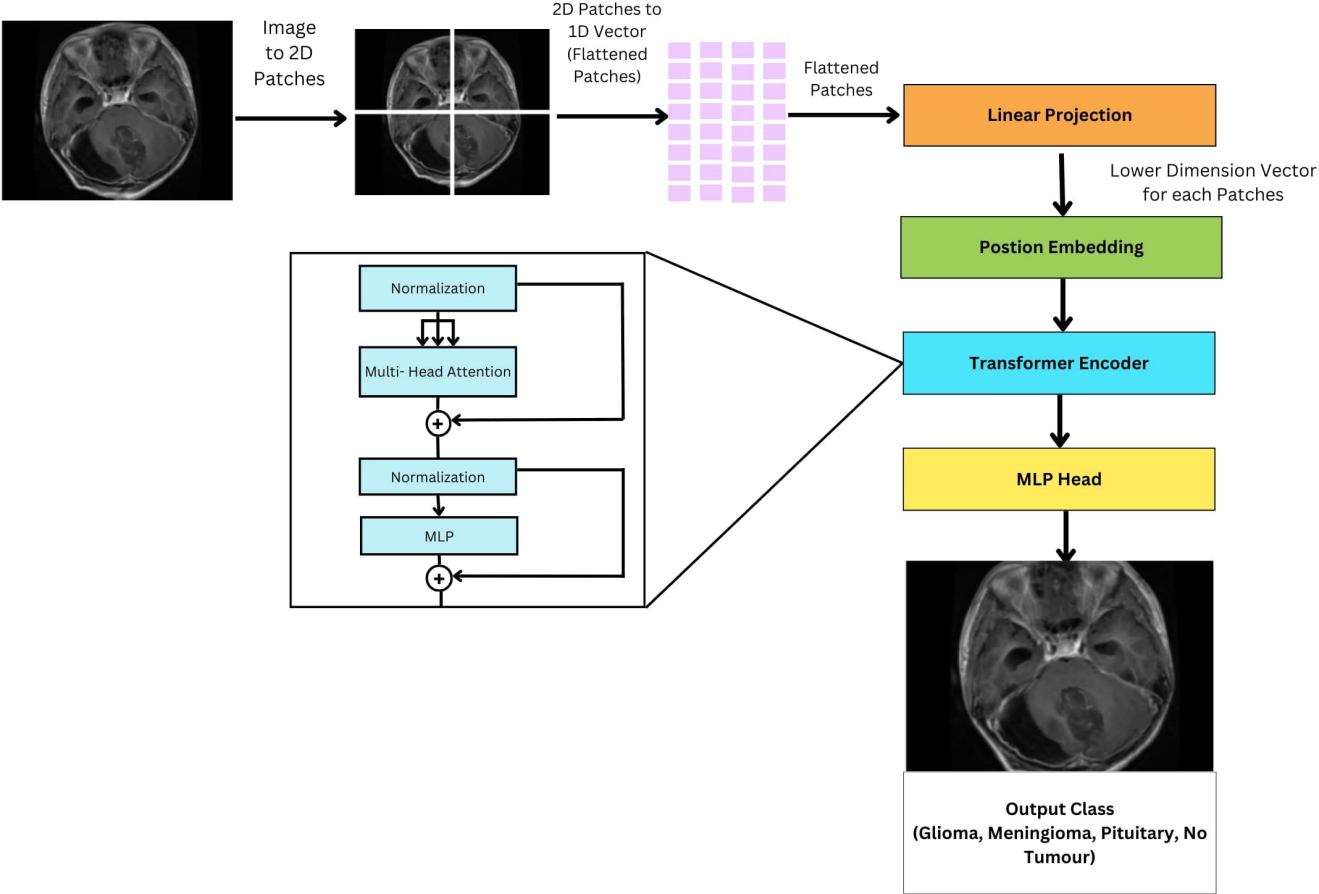
General architecture of vision transformer for multi-class brain tumor classification task using MRI scan imagery.

ViTs is an encoder-only transformer architecture without a decoder for image classification; see **Figure 10**. They utilize self-attention to understand the relationships between different parts of an image. The initial step involves the segmentation of the image into discrete, smaller regions known as patches. These patches are subsequently transformed into a linear sequence of tokens, each representing a distinct portion of the image. This transformation from a 2D image to a 1D sequence helps the model to understand and process these individual components of the image. The flattened patches undergo linear projection to transform them into lower dimensional vectors, preserving important features and relationships. To retain spatial information within the flattened sequence, additional “position embeddings” are incorporated, each encoding the original location of its corresponding patch within the image. The transformed patches enter the transformer encoder. Its self-attention layers and feedforward networks work together so that each patch can learn from the others, making the model aware of both localized features and larger patterns within the image. Unlike traditional transformers used in natural language processing, ViTs lack a decoder and instead feature an additional Linear layer called the MLP head (Multi-layer Perceptron) for final classification. This architectural difference reflects the focus of ViTs on extracting meaningful features and understanding spatial relationships within images for tasks like image classification and object detection. The encoder in a ViT leverages self-attention to achieve this goal, making it suitable for computer vision tasks.

#### 2.6.2 ViT model encoder

In a ViT, encoder operates in a multi-stage process. First, normalization ensures consistent data for smooth training. Then multi-head self-attention on each image patches to analyze spatial relationships. Then a normalization and MLP to refine and calculate more intricate patterns in the imag.e

Normalization (layer normalization), the input embeddings are first normalized using layer normalization. This step ensures that the input features have a consistent scale across different dimensions, which can improve the stability and convergence of the model. Where *γ* is learnable parameters for scaling and β is learnable parameters for shifting. *µ* is the mean of the input vector and *σ* ^2^ is the variance of the input vector x. A small constant *ε* is added for numerical stability.


$$LayerNorm\left(\text{x}\right)=\text{γ}\left(x-\text{µ}/\sqrt{{\text{σ}}^{2}+\text{ ϵ}}\right)+\text{ β}$$

Next is multi-head attention here after normalization, the normalized embeddings are passed through the multi-head attention mechanism. This mechanism computes attention scores between each pair of elements in the input sequence using learned weight matrices W_q_, W_k_, W_v_, and to the obtained weights the softmax function is applied. Where, Q is query, K is key, and V is value of matrices. The dimensionality of the key vectors is d_k_. ViT models use the multi-head self-attention to extract multifaceted information from the input sequence. Each attention head, denoted as head_i_, operates independently. It calculates attention scores by multiplying the query (W_i_^Q^) and key (W_i_^K^) weight matrices with the transformed input sequence. These scores represent the relevance of each element in the sequence to the current one. Subsequently, the scores are normalized using a softmax function, resulting in attention weights. These weights are then used to compute attention-weighted sums of the value vectors (W_i_^V^) from the input sequence. Finally, the outputs from all heads (head_i_) are concatenated and linearly transformed using the output projection matrix (W°.) to produce the final multi-head attention output. Where head_i_ represents the output of the i-th attention head. W_i_^Q^ is weight matrices for the query, W_i_^K^ is weight matrices for the key and W_i_^V^ is the weight matrices for the value projections of the i-th attention head respectively. The output projection matrix is represented by W°.


$$\text{Attention }\left(\text{Q},\text{ K},\text{ V}\right)=\text{softmax}\left({\text{QK}}^{\text{t}}/\surd {\text{d}}_{\text{k}}\right)*\text{ V}$$

$$\text{MultiHead}\left(\text{Q},\text{K},\text{V}\right)=\text{Concat}\left({\text{head}}_{1},\ldots .,{\text{head}}_{\text{h}}\right){\text{W}}^{\text{o}}\;$$

$$\text{head}=\text{Attention }\left({{\text{QW}}^{\text{Q}}}_{\text{i}},{{\text{KW}}^{\text{K}}}_{\text{i}},{{\text{VW}}^{\text{V}}}_{\text{i}}\right)$$

This step helps the model to capture dependencies and relationships between different parts of the input sequence.

Following the multi-head attention layer, the model incorporates a Layer Normalization step. This technique normalizes the activations of the network, ensuring they maintain a consistent distribution. This promotes stable gradients during training, which ultimately leads to improved model convergence and generalization performance.

Following the multi-head attention layer, the model utilizes a MLP to further refine the extracted features. This MLP, essentially a feed-forward neural network (FFNN), consists of two consecutive linear transformations separated by a non-linear activation function, typically ReLU. The purpose of this MLP is to extract even more complex patterns from the features identified by the multi-head attention. By applying these additional transformations, the model is able to learn higher level representations within the data. Where W1, W2 are the weight matrices of vectors of MLP and b1, b2 are the bias vectors of the MLP.


FFNN(x)=ReLU(xW1 + b1)W2 + b2

#### 2.6.3 Fine-tuning vision transformers

Unlike traditional fine-tuning approaches where only the final layers are modified, the FTVT model introduces a custom classifier head consisting of BN, dense layers, ReLU activation, and Dropout layers. This structural change enables the model to learn task-specific representations directly from the data. By incorporating task-specific layers, the FTVT model can adapt more flexibly and effectively to various tasks compared to standard fine-tuning approaches. These modifications allow the model to capture and leverage task-specific features, leading to improved performance and generalization. The introduction of structural changes in the FTVT model helps mitigate the risk of overfitting, especially on smaller datasets. The added regularization layers, such as dropout, prevent the model from memorizing noise in the training data, leading to better generalization performance. Leveraging pre-trained weights from the original ViT model, the FTVT model benefits from transfer learning while still retaining the flexibility to adapt to task-specific requirements. This approach combines the strengths of pre-training with the adaptability of task-specific fine-tuning, resulting in a more efficient and effective learning process.

The ViT models, especially the pretrained versions, such as ViT-b16, ViT-b32, ViT-l16, and ViT-l32, incorporate a Conv2d layer referred to as “conv_proj.” This layer is essential for computational efficiency; it reduces the number of parameters in the input image, that is, dimensionality reduction prior to it being fed into the computationally intensive transformer encoder layers. The input data format, 32 samples, three channels (RGB), and image size of 224 × 224 pixels. This information feeds into subsequent processing stages like patching and embedding. The pre-trained ViT model is fine-tuned by initializing it for the specific task. During training, a batch size of 16 images is used alongside the Adam optimizer with a learning rate of 1e−4. The Cross Entropy Loss function then evaluates the model’s performance on each batch, guiding parameter updates through the Adam optimizer to improve accuracy.


$$\text{Adam Optimizer }\left(\text{Optimizer}\right){\text{ θ}}_{\text{ t}+1}={\text{θ}}_{\text{t}}\;–{\;\epsilon *\;\text{m}}_{\text{t}}\;/\;\left(\surd {\text{v}}_{\text{t}}+\text{ η}\right)$$

This equation defines how the Adam optimizer updates the model’s weights (θ) during training. The learning rate (η) determines how much the weights are adjusted in each step. The terms m_t_ and v_t_ keep track of past changes to help the optimizer learn efficiently, and ϵ is a small value to avoid division by zero errors.


$$\text{Cross Entropy Loss}\left(\text{Loss Function}\right)=\;-1\;*\;\sum_{\text{i}}^{\text{N}}\text{yi }*\text{ log}\left(\hat{\text{y}}\text{i}\right)\;$$

The above equation represents the cross-entropy loss function, which is used during training to measure how far off the model’s predictions are from the actual labels. It considers the number of samples (N), the true labels (yi), and the predicted probabilities (ŷi) to calculate the error.

**Figure 11:**
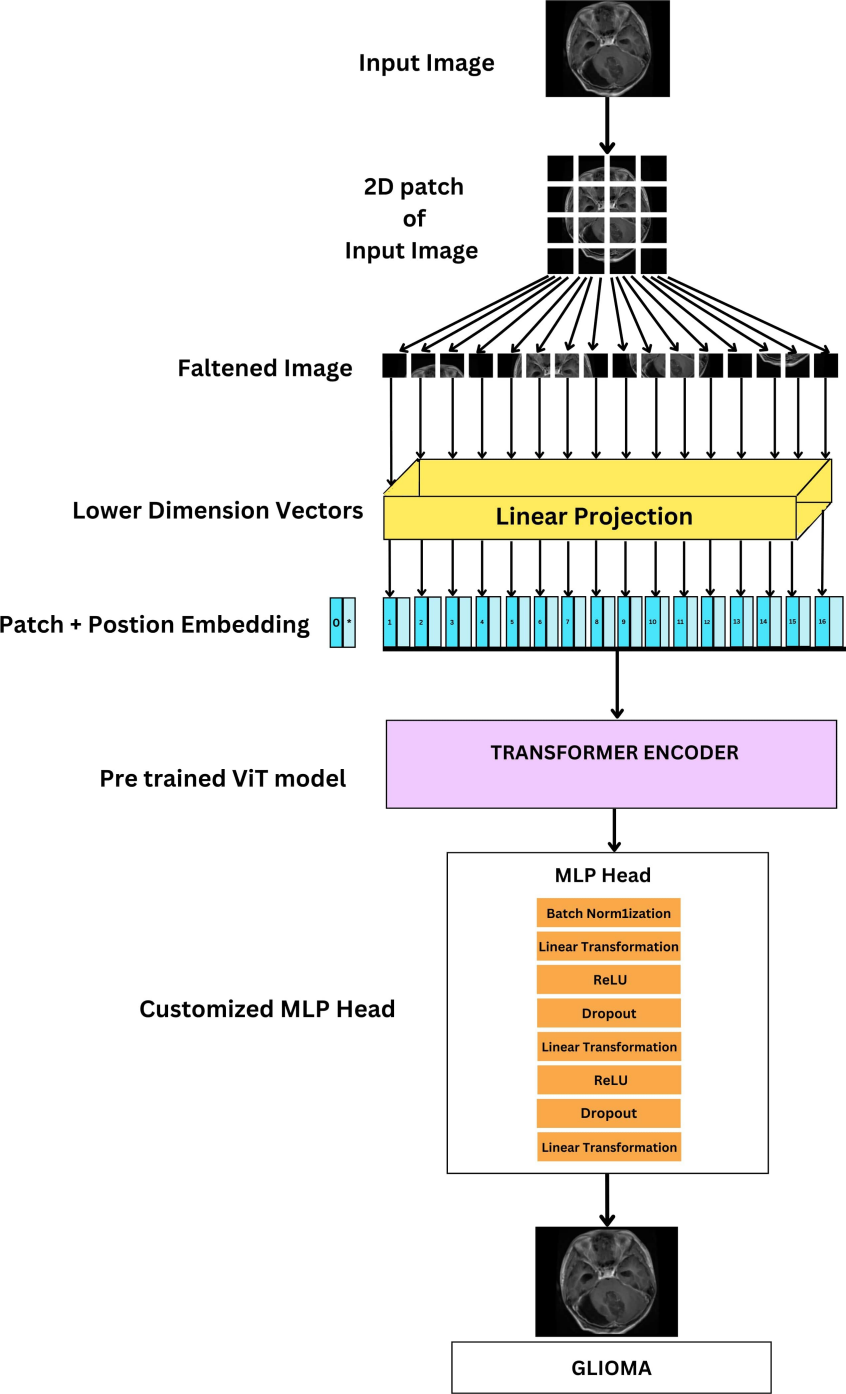
Architecture of FTVT for multi-class brain tumor classification task using MRI scan imagery.

The training process consists of iterating through the dataset for a total of 10 epochs, during which the model’s classifier head, integrated within the pretrained ViT feature extractor, is trained using the training dataset. The original classifier head is replaced with a custom sequence of layers, shown in **Figure 11** which includes BN, Linear (Dense), ReLU activation, and Dropout layers, thus making it a FTVT Model.

BN layer, Normalizes activations within each batch. This equation normalizes activations across each batch, improving stability and training speed. Where, μ is mean of the batch, σ₂ is variance of the batch and ϵ is small constant to prevent division by zero.


$$BN\left(x\right)\;=\;\left(x\;-\;\text{µ}\right)\;/\surd \left(\;{\text{σ}}^{2}+\;\text{ϵ}\right)$$

This layer uses the ReLU activation function. ReLU helps the model learn complex relationships by introducing non-linearity. It simply keeps positive values unchanged and turns negative values to zero.


RELU(x) = max(0, x) 

The Dropout regularization layer randomly drops neurons to prevent overfitting. This equation randomly sets activations to zero during training, preventing overfitting. Where *p* is the Dropout probability.


Dropout=x(with probability p) or 0 (otherwise)

The linear transformation layer here performs linear transformation on the input vector using weights (W) and biases (b).


Linear(x, W, b) = W * x + b 

These additional layers make the model better at learning intricate patterns, leading to improved overall performance. The final layer’s size is adjusted to match the number of brain tumor types that are being classified (four: glioma, tumor, meningioma tumor, no tumor, and pituitary tumor). The output from this modified section predicts the probability of each class, enabling accurate classification of brain tumor images.


X' = ReLU(BathNorm(W2 * Dropout(ReLU(BatchNorm(W1 * X)))))

The above equation represents the modified classifier head. It describes the final layers responsible for classification. Where X is input feature vector; it can have 768, 1,024 dimensions for B and L variants used in linear transformation. W1, W2 are weight matrices of Linear layers. BatchNorm, Dropout, and ReLU are BN layer, Dropout layer, and Rectified Linear Unit activation function.

### 2.7 Fine-tuned vision transformers models

#### 2.7.1 FTVT-b16

The architecture of the ViT-b16 model, representing the ViT with a “B” variant and 16 layers, comprises a total of 12 transformer blocks. Pretrained on the ImageNet dataset with over a million images, ViT-b16 undergoes fine-tuning using the Adam optimizer with a learning rate of 1e−4 and the Cross Entropy Loss function across 10 epochs. During this process, the parameters of both the pre-trained ViT-b16 model and the additional layers are updated through back propagation. The input image size is set at 224 × 224 pixels. To enhance its classification performance, the ViT-b16 model’s classifier head incorporates extra layers including BN, Linear, ReLU, and Dropout layers, thus making it FTVT-b16. These layers are designed to improve the model’s ability to identify crucial features relevant to the target categories. Their parameters are randomly initialized and trained concurrently with the existing parameters of the ViT-b16 model. The input features for these additional layers correspond to the model’s output size, which is 768. The sequence of added layers begins with BN, followed by a linear transformation layer, ReLU activation function, and a Dropout layer with a rate of 0.5 to prevent overfitting. Subsequently, another linear transformation layer reduces the feature dimensionality to 512, followed by ReLU activation and another Dropout layer with the same rate for regularization. Finally, the feature vector is transformed into the output space via a Linear layer, determining the number of output classes representing brain tumor categories.

#### 2.7.2 FTVT-b32

The architecture of the ViT-b16 model, representing the Vision Transformer with a “B” variant and 16 layers, comprises a total of 12 transformer block has been trained on the ImageNet dataset (millions of images). It is fine-tuned for brain tumor classification. Adam optimizer (lr = le−4) and Cross-Entropy Loss are used over 10 epochs to adjust model parameters. During this process, backpropagation optimizes both pre-trained and additional layer parameters. For input, images were resized to a standard 224 × 224 pixel. To enhance its classification performance, the ViT-b16 model’s classifier head incorporates extra layers including BN, Linear, ReLU, and Dropout layers thus making it FTVT-b16. This set of layers is specifically engineered to enhance the model’s capacity to discern critical features that are directly linked to the target categories. Their parameters are randomly initialized and trained concurrently with the existing parameters of the ViT-b16 model. The input features for these additional layers correspond to the model’s output size, which is 768.The additional layers begins with BN for stable training. This is followed by a linear transformation layer where feature dimensionality is reduced from 768 to 512 and a ReLU activation function to introduce non-linearity. A Dropout layer with a rate of 0.5 is then incorporated to prevent overfitting. Finally, the feature dimensionality is reduced to 256 through another linear transformation, followed by ReLU activation and another Dropout layer (0.5) for regularization. Finally, the feature vector is transformed into the output space via a Linear layer, determining the number of output classes representing brain tumor categories.

#### 2.7.3 FTVT-l16

The ViT-l16 model, identified by its “L” variant and 16 layers, consists of 12 transformer blocks within its architecture had been trained on the ImageNet dataset (millions of images). It is fine-tuned for brain tumor classification. Adam optimizer (lr = le-4) and Cross-Entropy Loss are used over 10 epochs to adjust model parameters. During this process, backpropagation optimizes both pre-trained and additional layer parameters. For input, images were resized to a standard 224 × 224 pixel. To enhance its classification performance, the ViT-l16 model’s classifier head incorporates extra layers including BN, Linear, ReLU, and Dropout layers, thus making it FTVT-l16. Their parameters are randomly initialized and trained concurrently with the existing parameters of the pretrained ViT-l16 model. The input features for these additional layers correspond to the model’s output size, which is 1024. The additional layers begin with BN for stable training. This is followed by a linear transformation layer where feature dimensionality is reduced from 1024 to 512 and a ReLU activation function to introduce non-linearity. A Dropout layer with a rate of 0.5 is then incorporated to prevent overfitting. Finally, the feature dimensionality is reduced to 256 through another linear transformation, followed by a ReLU activation and another Dropout layer (0.5) for regularization. Finally, the feature vector is transformed into the output space via a Linear layer, determining the number of output classes representing brain tumor categories.

#### 2.7.4 FTVT-l32

The ViT-l32 model, denoted by its “L” variant and 32 layers, comprises 24 transformer blocks. It has been trained on the ImageNet dataset (millions of images). It is fine-tuned for brain tumor classification. Adam optimizer (lr = le-4) and Cross-Entropy Loss are used over 10 epochs to adjust model parameters. During this process backpropagation optimizes both pre-trained and additional layer parameters. For input, images were resized to a standard 224 × 224 pixel. To adapt the model for the task, additional layers are introduced into its classifier head. These layers, include Batch Normalization (BN), Linear, ReLU, and Dropout layers, make up the FTVT-b32 model. With randomly initialized parameters, these layers are trained alongside the existing parameters of the ViT-l32 model. The input features for these layers match the model’s output size of 1024. The additional layers begin with BN for stable training. This is followed by a linear transformation layer where feature dimensionality is reduced from 1024 to 512 and a ReLU activation function to introduce non-linearity. A Dropout layer with a rate of 0.5 is then incorporated to prevent overfitting. Finally, the feature dimensionality is reduced to 256 through another linear transformation, followed by a ReLU activation and another Dropout layer (0.5) for regularization. Finally, the feature vector is transformed into the output space via a Linear layer, determining the number of output classes representing brain tumor categories.

### 2.8 Experimental procedure

The first step in data preprocessing involved standardizing the brain MRI images by resizing them to a uniform dimension. This resizing to 256 × 256 pixels was chosen to balance sufficient resolution for accurate brain tumor diagnosis and efficient training times. Next, feature extraction was carried out using deep learning models on the preprocessed MRI images. Our approach utilized novel FTVTs – including FTVT-b16, FTVT-b32, FTVT-l16, and FTVT-l32 for brain tumor classification. These were compared against established deep learning models such as ResNet50, MobileNet-V2, and Efficientb0. The FTVT models were trained on the preprocessed images using suitable optimization techniques and loss functions. For evaluation and prediction, a separate test set of MRI images was used to assess the trained model’s performance. Metrics such as accuracy, precision, recall, and F1-score were calculated to evaluate the model’s effectiveness. Finally, the trained model was applied to new MRI scans to predict brain tumors, providing a probability score for each class, with the highest score indicating the predicted class.

**Figure 12:**
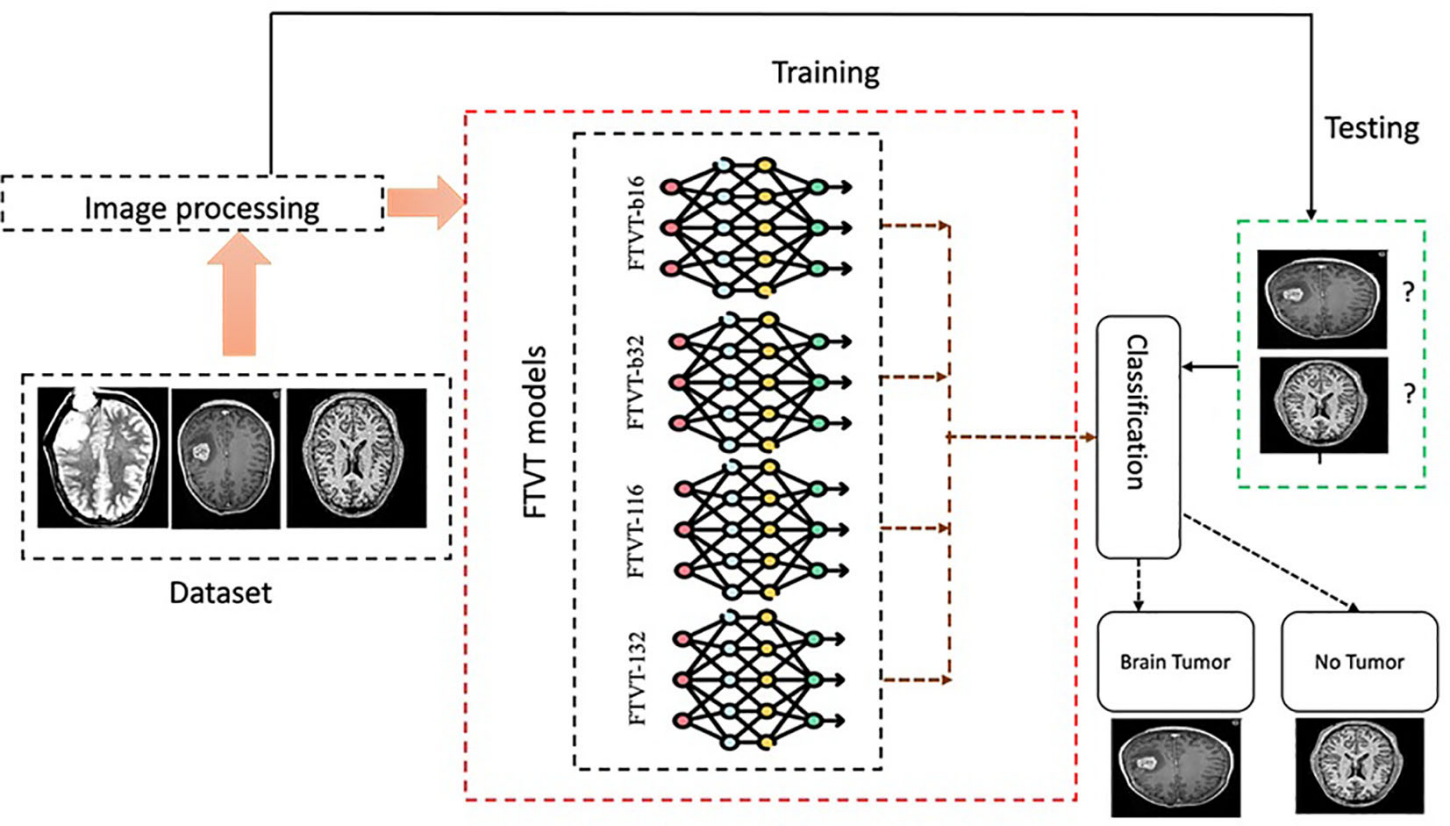
Experimental procedure flowchart.

The following points outline the pipeline for brain tumor classification, as shown in **Figure 12**:

- Specify the path to the dataset directory.
- Convert hexadecimal files to images: this function transforms hexadecimal data into images, ensuring each array has 16 columns.
- Reshape the data into square images and save them as JPEG files.
- Apply Gaussian blur to the images using OpenCV.
- Preprocess and load dataset: utilize ImageDataGenerator to load images from the directory.
- flow_from_directory loads images, resizes them to 256 × 256, and creates batches.
- Train-Test Split: divide the dataset into training and testing sets, normalizing the image data.
- Define and compile model: load the model without its top layers, add a global average pooling layer, and a dense layer for classification.
- Freeze the base model layers and compile the model.
- Compute class weights and train model: calculate class weights to address class imbalance and train the model with the training data using the computed weights.
- Confusion matrix and classification report: Evaluate the model on the test data, generating and printing a classification report and confusion matrix.

### 2.9 Evaluation metrics

In this study, we utilized several key metrics to evaluate the performance of our models in the task of brain tumor classification. These metrics provide valuable insights into the effectiveness of the models in making accurate predictions and can help in determining their overall reliability.

#### 2.9.1 Accuracy

Accuracy measures the proportion of correctly predicted samples out of the total number of samples in the dataset. It provides an overall assessment of the model’s performance across all classes. A higher accuracy indicates a better ability of the model to classify samples correctly.


$$\text{Accuracy}=\frac{\text{TN}+\text{TP}}{\text{TP}+\text{TN}+\text{FP}+\text{FN}}$$

#### 2.9.2 F1 Score

The F1 score is the harmonic mean of precision and recall. It considers both false positives and false negatives and is particularly useful when dealing with imbalanced datasets. The F1 score provides a balance between precision and recall, with higher values indicating better performance.


$$F1\;Score=\;2\times \frac{Precision\;x\;Recall}{Precision+Recall}$$

#### 2.9.3 Precision

Precision measures the proportion of true positive predictions out of all positive predictions made by the model. It indicates the model’s ability to accurately identify relevant samples within a class, minimizing false positives. Higher precision values signify fewer false positives and higher confidence in positive predictions.


$$Precision=\frac{TP}{TP+FP}$$

#### 2.9.4 Recall

Recall, also known as sensitivity or true positive rate (TPR), measures the proportion of true positive predictions out of all actual positive samples in the dataset. It assesses the model’s ability to capture all relevant samples within a class, minimizing false negatives. Higher recall values indicate fewer false negatives and better coverage of positive samples.


$$Recall=\frac{TP}{TP+FN}$$

## 3 Results

Using Google’s proprietary Kaggle platform’s notebook the proposed experiment was conducted. Within Kaggle, the Kaggle Kernel acts as a free Jupyter notebook server with GPU integration. This allows cloud computing resources to be used instead of relying solely on local machines. Two GPU T4 instances each with a memory amount of 15 GB for handling large image datasets and a RAM size of 29 GB were used. The coding was performed in Python, the PyTorch library was used for building Deep learning and FTVT models and the Matplotlib library to visualize the results.

### 3.1 Deep learning models

**Figure 13:**
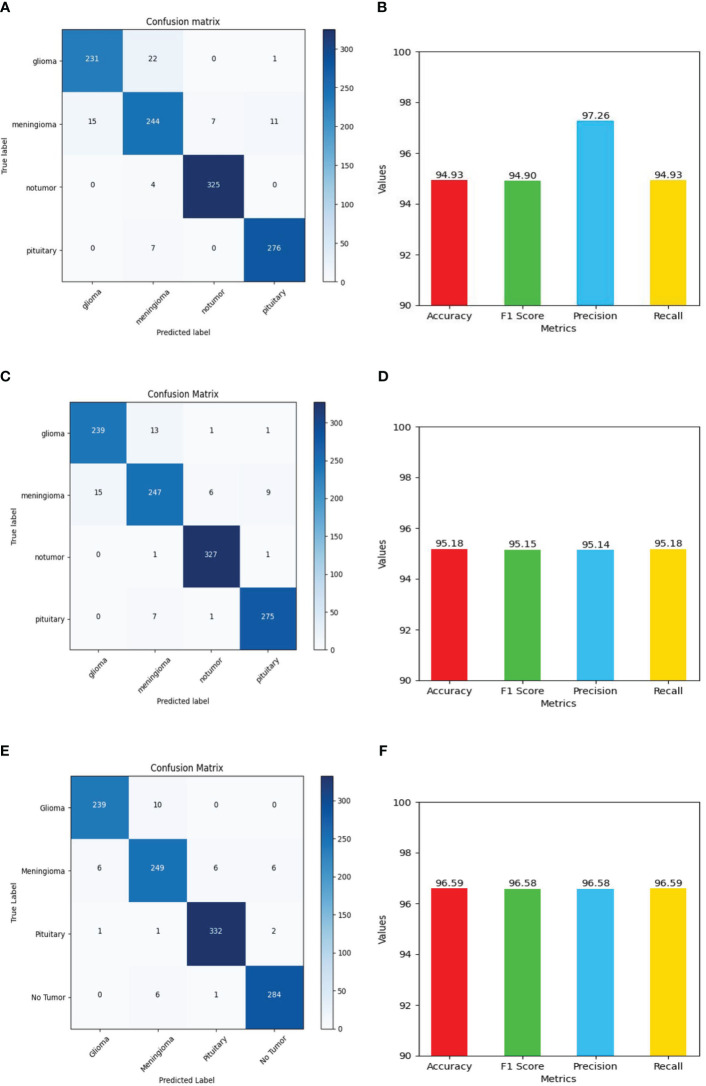
Shows values of evaluation metrics (accuracy, F1 Score, precision, recall) and confusion matrix for Deep Learning models for multi-class brain tumor classification task using MRI scan imagery: **(A, B)** confusion matrix and evaluation metrics of MobileNet-V2; **(C, D)** confusion matrix and evaluation metrics of EfficientNet-B0; **(E, F)** confusion matrix and evaluation metrics of ResNet-50.

**Figure 14:**
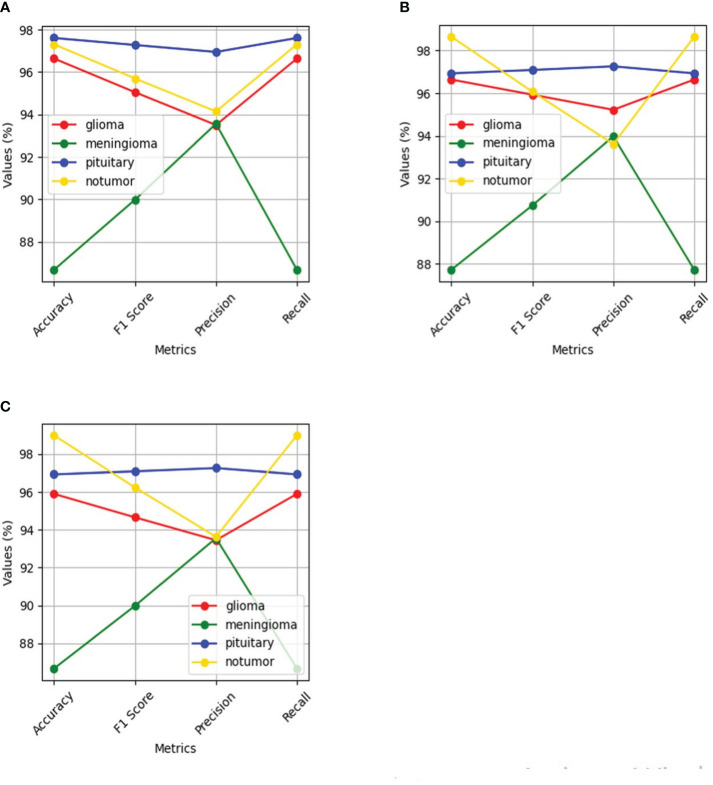
Shows performance of the four classes namely glioma, meningioma, pituitary and no tumor for the three deep learning models for Multi-Class Brain tumor classification task using MRI scan imagery: **(A)** ResNet-50; **(B)** EfficientNet-B0; **(C)** MobileNet-V2.

The performance of the Deep learning model using the evaluation metrics (accuracy, F1 score, precision, and recall) for individual model with its confusion matrix is shown in **Figure 13** and across tumor classes (glioma, meningioma, pituitary, and no tumor) using **Table 2** and **Figure 14**.

**Table 2:** Provides an in-depth analysis of performance of the four classes namely glioma, meningioma, pituitary, and no tumor for the three deep learning models for multi-class brain tumor classification task using MRI scan imagery.

Model	Class	Evaluation metrics				
		Accuracy (%)	F1 score (%)	Precision (%)	Recall (%)	Images misclassified
ResNet-50	Glioma	96.64	95.05	93.50	96.64	11
	Meningioma	86.67	89.98	93.56	86.67	38
	Pituitary	97.61	97.28	96.95	97.61	8
	No tumor	97.31	95.70	94.14	97.31	8
EfficientNet-B0	Glioma	96.64	95.93	95.22	96.64	9
	Meningioma	87.72	90.74	93.98	87.72	35
	Pituitary	96.93	97.09	97.26	96.93	9
	No tumor	98.65	96.07	93.61	98.65	4
MobileNet-v2	Glioma	95.90	94.66	93.45	95.90	11
	Meningioma	86.67	89.98	93.56	86.67	38
	Pituitary	96.93	97.09	97.26	96.93	9
	No tumor	98.99	96.24	93.63	98.99	3

#### 3.1.1 ResNet-50

The ResNet-50 **Figures 13E, F** achieved values ranging from 96.58% to 96.59% for all the metrics. Particularly showed high values in accuracy and recall. From **Figure 14A** and **Table 2**, it can be observed that the accuracy of the pituitary class is highest with 96.6% and accuracy of the meningioma class is lowest with 86.6%, while the glioma and no tumor classes acquired 97.6% and 97.3% respectively. We can observe that the recall has a similar pattern with highest class being pituitary (97.6%), lowest being meningioma (86.6%) followed by glioma (96.6%) and no tumor (97.3%). The F1 score for meningioma (89.9%), glioma (95%), no tumor (95.7%), and pituitary (97.2%) is in increasing order. The precision values of glioma and meningioma coincide at 93.5% and no tumor, pituitary acquired 94% and 96%. Overall, ResNet-50 performed well in detecting pituitary class with all the evaluation metrics being highest than others.

#### 3.1.2 EfficientNet-b0

The EfficientNet-b0 **Figures 13C, D** model showed consistent performance across all the metrics, with the values ranging from 95.14% to 95.18%. The F1 values indicate its good performance in correctly identifying positive cases while minimizing false positive predictions. The no tumor class obtained highest accuracy and recall which is 98.6%, but the precision for no tumor is least with 93.6% and it acquired a moderate F1 score of 96% from **Figure 14B** and **Table 2**. The accuracy, recall, and F1 score of meningioma is least with 87.7%, 87.7%, and 90.7%, respectively, with 93.9% recall, which is slightly higher than no tumor. This indicates that the EfficientNet-b0 model and ResNet-50 model are similar as both the models performed well in classifying no tumor and could not identify meningioma type of brain tumors perfectly. EfficienetNet-b0 performed moderately in classifying glioma and pituitary classes with an accuracy of 96% and precision, recall, f1 score all greater than 95%.

#### 3.1.3 MobileNet-v2

The MobileNet-v2 **Figures 13A, B** achieved values ranging from 94.92% to 95.26% across all the metrics. Its high precision value indicates a low false-positive rate and a high proportion of correctly identified positive instances among all predicted positive instances. As we can observe, the graphs of MobileNet-v2 and EfficientNet-b0 models **Figure 14B** and **Table 2** are similar in pattern indicating that the performance of these two models is almost similar. As meningioma is detected by EfficientNet-b0 with least accuracy, the MobileNet-v2 has also acquired least accuracy for meningioma class which is 86.6%.The precision, recall, and f1 score of this class are 93.5%, 86.65%, and 89.9%, respectively. The highest accuracy is again obtained for no tumor (98.9%) class while the precision, recall and f1 score of this class are 93.6%, 98.9%, and 96.4%.The other classes which are pituitary and glioma has the accuracy, precision, recall, and f1 score values of 95.9%, 93.4%, 95.9%, 94.6% and 96.9%, 97.2%, 96.9%, 97%, respectively.

### 3.2 FTVT models

**Figure 15:**
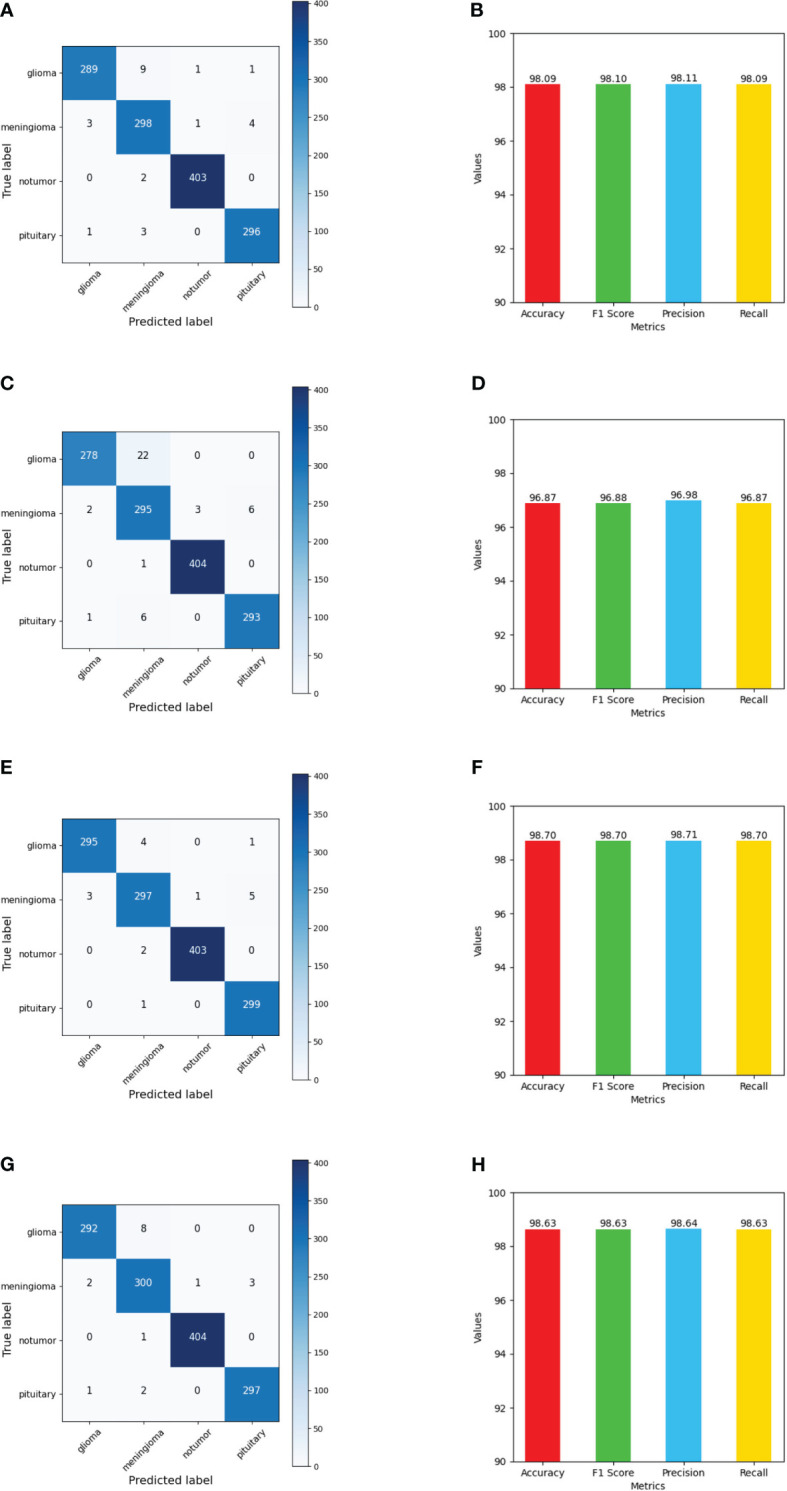
Shows values of evaluation metrics (accuracy, F1 Score, precision, recall) and confusion matrix for fine-tuned vision transformer models for multi-class brain tumor classification task using MRI scan imagery: **(A, B)** confusion matrix and evaluation metrics of FTVT-l16; **(C, D)** confusion matrix and evaluation metrics of FTVT-b32; **(E, F)** confusion matrix and evaluation metrics of FTVT-l16; **(G, H)** confusion matrix and evaluation metrics of FTVT-l32.

The performance of the FTVT model using the evaluation metrics (accuracy, F1 score, precision, and recall) for each model along with the confusion matrix is shown in **Figure 15** and across tumor classes (glioma, meningioma, pituitary, and no tumor) using **Table 3** and **Supplementary Figure 1**.

**Table 3:** Provides an in-depth analysis of performance of the four classes, namely, glioma, meningioma, pituitary, and no tumor for the FTVT models for multi-class brain tumor classification task using MRI scan imagery.

Model	Class	Evaluation metrics				
		Accuracy (%)	Precision (%)	Recall (%)	F1 score (%)	Images misclassified
FTVT-b16	Glioma	98.85	98.63	96.33	97.47	11
	Meningioma	98.32	95.51	97.39	96.44	8
	Pituitary	99.31	98.34	98.67	98.50	4
	No tumor	99.69	99.51	99.51	99.51	2
FTVT-b32	Glioma	98.09	98.93	92.67	95.70	22
	Meningioma	96.94	91.05	96.41	93.65	11
	Pituitary	99.00	97.99	97.67	97.83	7
	No tumor	99.69	99.26	99.75	99.51	1
FTVT-l16	Glioma	99.38	98.99	98.33	98.66	5
	Meningioma	98.77	97.70	97.06	97.38	9
	Pituitary	98.03	99.67	98.67	98.84	2
	No tumor	99.77	99.75	99.51	99.63	1
FTVT-l32	Glioma	99.16	98.98	97.33	98.15	8
	Meningioma	98.70	96.46	98.04	97.24	6
	Pituitary	99.54	98.43	98.67	98.50	3
	No tumor	99.84	99.51	99.51	99.51	1

#### 3.2.1 FTVT-b16

FTVT-b16 model **Figures 15A, B** showed remarkable performance in all the evaluation metrics accuracy, F1 score, recall, and precision. It achieved values ranging from 98.09% to 98.10% for all the metrics. Its high accuracy and recall values ​​show how well it is able to classify true positive and true negative values in all categories. FTVT-b16 model **Supplementary Figure 1A** and **Table 3** across all classes, demonstrated an accuracy ranging from 98.85% to 99.69%. Particularly, for pituitary tumor classification, it demonstrated the highest accuracy of 99.31% among other two tumor classes. Its achieved precision values in the range of 95.51%–99.51%; it showed 98.63% for glioma, which is the highest and 95.51% in meningioma which is the lowest in the other two tumor classes. Recall and F1 score values are in the range of 96.33%–99.51%, where 98.67% recall and 98.50% F1 score in pituitary are highest among other tumor classes. For no tumor class, the FTVT-b16 achieved the highest values across all the metric consistently in the range of 99.51% to 99.69%.

#### 3.2.2 FTVT-b32

The FTVT-b32 **Figures 15C, D** model performed consistently across all the metrics from values ranging from 96.87% to 96.98%, but comparatively scored lowest among the other FTVT models. FTVT-b32 **Supplementary Figure 1B** and **Table 3** model showed highest values for all metrics in no tumor class ranging from 99.26% to 99.75%, which is higher than FTVT-b16. Its accuracy is ranging from 96.94% to 99.69% and scored highest in pituitary tumor class with 99.00%, which is highest in all the other tumor classes. Its precision, recall and F1 score values are ranging from 91.05% to 99.26%, 92.67% to 99.75%, and 93.65% to 99.51%. In glioma tumor class, it scored highest precision of 98.93%; in pituitary, it achieved 97.67%, 97.83% recall, F1 score which are higher than other tumor classes, but showed consistently lower performance for meningioma tumor class. Except for no tumor class it demonstrated slightly lower performance than FTVT-b16.

#### 3.2.3 FTVT-l16

FTVT-l32 **Figures 15E, F** model achieved accuracy ranging from 98.62% to 98.63% having the second highest values among all models. The FTVT-l16 model **Figures 15E, F** performed remarkably by achieving the highest values in all the metrics among all the models ranging from 98.63% to 98.64%. The FTVT-l16 model **Supplementary Figure 1C** and **Table 3** exhibited superior performance with accuracies ranging from 98.03% to 99.77% across all classes. For the glioma tumor class, it achieved 99.38% accuracy, which is highest in all other tumor classes. FTVT-l16 consistently achieved precision, recall, and F1 scores values ranging from 97.02% to 99.75%. It achieved the highest values of 99.67%, 98.67%, and 98.84% for precision, recall, F1 score, which higher than all tumor classes, followed by in glioma and meningioma. In no tumor class, it achieved the highest values across all the metrics.

#### 3.2.4 FTVT-l32

The FTVT-l32 model **Figures 15G, H**, **Supplementary Figure 1D**, and **Table 3** also demonstrated excellent performance, with accuracies ranging from 98.70% to 99.84%. The highest in pituitary with 99.54% followed by glioma 99.16%, which is higher than meningioma 98.70%. Its precision values are in the range of 96.46%–99.51%, with 98.98% in glioma which is higher than the other two tumor classes. Its precision and recall is in the ranges of 97.24% to 99.51%, with 98.67%, 98.04% recall in pituitary and meningioma and 98.15%, 98.50% F1 score in glioma, pituitary tumor classes, with 99.84% accuracy in no tumor class which is highest among all the models.

## 4 Discussions

This study investigates brain tumor classification through preprocessing data, fine-tuning pre-trained models, and evaluating performance metrics such as accuracy, precision, recall, and F1 score. By analyzing model performance for each tumor class and deploying models on unseen data, it aims to enhance diagnostic accuracy and clinical applicability in medical imaging.

In this study, the FTVT models and the established deep learning models—ResNet50, MobileNet-V2, and EfficientNetb0—are compared, primarily focusing of FTVT models. FTVT models, including FTVT-B16, FTVT-B32, FTVT-L16, and FTVT-L32, consistently outperform ResNet50, MobileNet-V2, and Efficientb0 in brain tumor classification tasks across all metrics, as shown in **Supplementary Figure 2**. Additionally, **Supplementary Figure 3** highlights FTVT models’ superior performance in accuracy, precision, recall, and F1-score across tumor classes—glioma, meningioma, pituitary, and no tumor. This superiority can be attributed to the custom classifier head introduced in FTVT models, allowing for task-specific representations directly from the data. Also FTVT models incorporate attention mechanisms, enabling them to effectively capture intricate patterns and dependencies within medical imaging data.

The FTVT models’ exceptional performance metrics demonstrate their ability to distinguish between distinct forms of brain tumors. With accuracy levels reaching 98% and precision, recall, and F1-score values continuously exceeding 96%, these models demonstrate extraordinary precision in their predictions, which is critical for precise clinical diagnosis and treatment stratification. This outstanding performance can be attributed to the sophisticated architectures of the FTVT models, which were rigorously fine-tuned utilizing optimization techniques. Furthermore, the addition of extra layers considerably contributes to the increased precision reported across all FTVT models, reaching 99.75%. Furthermore, these layers improve the models’ ability to classify brain tumors by capturing fine information in medical imaging.

Based on the comparison of the models’ performance values provided in the results section, **Supplementary Figure 2**, we can see that FTVT-l16 achieved the highest overall accuracy of 98.70%, followed by FTVT-l32 with an accuracy of 98.63%, indicating the model’s overall correctness in predicting tumor types, reflecting the proportion of correctly classified instances out of the total evaluated. Both models demonstrated exceptional precision scores, demonstrating that they can properly classify tumor types across the board. Furthermore, FTVT-l16 had a significantly higher recall rate for glioma tumors (98.33%) than FTVT-l32 (97.33%) in the same category. High recall values across all models indicate their efficiency in catching true positives, which improves their capacity to detect relevant tumor types. In contrast, the FTVT-b32 and FTVT-b16 models had commendable precision and recall values, with FTVT-b16 having a slightly higher recall rate for meningioma tumors (97.39%) than FTVT-b32 (96.41%). The F1-score, FTVT-l16, and FTVT-l32 models achieved high scores of 98.66% and 98.54%, respectively, demonstrating balanced performance in terms of precision and recall. While the FTVT-b32 and FTVT-b16 models had respectable precision and recall values, their F1-scores were slightly lower than the FTVT-l16 and FTVT-l32 models, indicating a little less balanced performance in tumor classification. Overall findings indicate that the FTVT-l16 and FTVT-l32 models perform consistently across all tumor types, but the FTVT-b16 and FTVT-b32 models excel at identifying specific tumor types.

The confusion matrices provided a detailed breakdown of each model’s performance, revealing where certain tumor types might pose challenges. Despite slight variations, all models showcased exceptional accuracy, precision, recall, and F1-score values. For instance, in FTVT-b16, with an overall accuracy of 98.09%, minimal misclassifications were observed across all tumor classes: 11 misclassified images in glioma out of 300, eight in meningioma out of 306, two in no tumor out of 405, and four in pituitary out of 300. Similarly, FTVT-l16 achieved an impressive overall accuracy of 98.70%, with slight misclassifications across tumor classes: five in glioma, nine in meningioma, fwo in no tumor, and one in pituitary. However, FTVT-b16 and FTVT-l16 outperformed both FTVT-b32 and FTVT-l32 models in terms of overall accuracy and lower misclassification rates, particularly noticeable in glioma and meningioma classes. The superior performance of B16 (FTVT-b16) and L16(FTVT-l16) models can also be attributed to their finer granularity in feature extraction and representation due to the smaller patch size utilized in these models compared to B32(FTVT-b32) and L32(FTVT-l32), that is, B16 is less than B32 and L16 is less than L32. This finer granularity allows B16 and L16 models to capture more intricate details in the medical images, leading to improved classification accuracy and lower misclassification rates.

The evaluation measures show that the FTVT models have distinct strengths when it comes to tumor type classification **Supplementary Figure 3**. For pituitary tumors and gliomas, FTVT-b16 shows excellent accuracy; however, for meningiomas, its accuracy is marginally lower. While FTVT-b32 performs better than FTVT-b16 in terms of overall accuracy, especially for no tumors, it exhibits greater misclassifications for gliomas and meningiomas. With balanced precision and recall across all classes, FTVT-l32 demonstrates remarkable accuracy for gliomas and no tumors, leading to fewer misclassifications. Likewise, FTVT-l16 attains exceptional precision for every class with negligible misclassifications, establishing it as a formidable candidate for precise tumor classification across the board. Overall, FTVT-l32 and FTVT-l16 stand out for their balanced performance and high accuracy, while the choice between models may depend on specific tumor classification priorities. When comparing all the model we observer L (L16, L32) variants are performing better than B(B16, B32) variants this because the variance in patch size between B and L types significantly impacts model performance. Smaller patch sizes in L models, like FTVT-l32 and FTVT-l16, capture finer details in medical images, potentially enhancing accuracy and reducing misclassifications. Consequently, this distinction likely contributes to the superior performance of L type models over their B type counterparts.

The remarkable ability of the FTVT models to correctly identify different kinds of brain tumors highlights their potential for use in clinical applications. The meticulous fine-tuning of these models, coupled with the incorporation of additional layers, has significantly enhanced their precision and recall values, crucial for reliable diagnosis and treatment planning. Despite slight variations in performance across different models, all FTVT variants demonstrated exceptional accuracy, precision, recall, and F1-score values, highlighting their robustness in tumor classification. Moving forward, further research into refining these models and addressing any remaining limitations could bolster their real-world applicability in medical settings. Overall, the findings of this study contribute to the growing body of evidence supporting the utility of machine learning models, particularly FTVT architectures, in enhancing medical image analysis and diagnostic capabilities.

In **Table 4**, various algorithms were employed to classify brain tumors, each exhibiting different performance measures. The FTVT models proposed in this study outperform all the exiting methodologies.

**Table 4:** The models and performance measures of the relevant work for brain tumor classification task.

S NO.	ALGORITHM USED	PERFORMANCE MEASURES
1	Custom CNN model (24)	accuracy-91.9%, recall-95.07%, precision- 94.81%, and F1-score:94.94%
2	Multi-stream 2D-CNN model (25)	mean-accuracy:88.82%, mean-specificity:92.17%, and mean-sensitivity:81.81%
3	Convolutional Neural Network based on Complex Networks (16)	accuracy 95.49%
4	DNN(Deep Neural Networks) (27)	accuracy 96.15%, precision 94.12%, F1-score 96.97%, and recall 100%
5	VGG16 (28)	accuracy 89%, sensitivity 87%, specificity 92%
6	CNN,VGG16,ResNet-50, and Inception V3 (14)	(%) Accuracy Recall Loss CNN 93.3 91.13 0.25 ResNet-50 81.10 81.04 0.85 VGG16 71.60 70.03 1.18 Inception V3 80.00 79.81 0.25
7	Fine tuned and pre trained ViTs and convolutional neural network models (23)	Accuracy Validation Loss Loss Accuracy (train) (test) R50-ViT-l16 0.98 0.90 0.03 0.0.05 ViT-l32 1.00 0.94 0.04 0.12 ViT-b16 0.99 0.97 0.01 0.10 ViT-b32 0.98 0.98 0.04 0.12 ViT-l16 0.98 0.97 0.08 0.15
8	ViT models (B-16, B-32, L-16, and L-32), (22)	All ViT models Accuracy Sensitivity Specificity 97.71 96.87 99.10
9	The proposed Approach	(%) Accuracy F1 Precision Recall FTVT-b16 98.1 98.1 98.1 98.1 FTVT-l16 96.9 96.9 97.0 96.9 FTVT-l32 98.7 98.7 98.7 98.7 FTVT-b32 98.6 98.6 98.6 98.6

## 5 Conclusion

This study presents a simple and detailed comparative analysis of various deep learning models (ResNet-50, EfficientNet-b0, and MobileNet-V2) and FTVT (FTVT-b16, FTVT-b32, FTVT-l16, and FTVT-l32). This work also differentiates the architecture of the models, which helps to provide a broader view. Introduction of new layers for optimization and hyperparameter tuning to prevent overfitting is an added advantage which provided high accuracy for both deep learning and FTVT models than previous studies **Table 4**, which shows the significance of the fine tuning performed in this study. A dataset containing a large number (7,053) of brain MRI scans, diverse preprocessing techniques such as data augmentation, normalization, and rigorous training are used for training the models. This study has achieved outstanding accuracies for brain tumor classification which helps professionals to detect and classify tumors in the early stages. The models are assessed with various evaluation metrics for better analysis and comparison individually and across each tumor class. The FTVT models achieved accuracies greater than other deep learning models ResNet-50, EfficientNet-b0, and MobileNet-V2. Also, FTVT models individually and across tumor classes outperformed these deep learning models. We see a trend of L variant performing better than B variant, a general trend observed in pretrained ViTs, (22). And also FTVT-l16, FTVT-b16 performed better than FTVT-l32, FTVT-b 32, which suggest that “16” models are more effective at capturing fine details. The decision of using L or B variant depends upon the computational resources available; B variant uses comparatively less resources than the L variant. With the observed results and thorough analysis, it is evident that the FTVT are robust and accurate proving their incredible role in medical image analysis.

## Data availability statement

The original contributions presented in the study are included in the article/**Supplementary Material**. Further inquiries can be directed to the corresponding author.

## Author contributions

CR: Conceptualization, Methodology, Writing – original draft. PR: Conceptualization, Formal analysis, Methodology, Writing – original draft. HJ: Formal analysis, Methodology, Writing – original draft. BA: Conceptualization, Funding acquisition, Methodology, Supervision, Validation, Writing – review & editing. MS: Investigation, Methodology, Visualization, Writing – review & editing. SA: Conceptualization, Project administration, Supervision, Validation, Writing – review & editing. AS: Investigation, Project administration, Resources, Supervision, Validation, Visualization, Writing – review & editing.

## References

[1] Asiri AA Shaf A Ali T Aamir M Usman A Irfan M. Multi-level deep generative adversarial networks for brain tumor classification on Magnetic Resonance Images. Intelligent Automation Soft Comput. (2023) 36:127–43. doi: 10.32604/iasc.2023.032391

[2] Villanueva-Meyer JE Mabray MC Cha S. Current clinical brain tumor imaging. Neurosurgery. (2017) 81:397–415. doi: 10.1093/neuros/nyx103 28486641 PMC5581219

[3] Ostrom QT Gittleman H Truitt G Boscia A Kruchko C Barnholtz-Sloan JS. CBTRUS statistical report: Primary Brain and other central nervous system tumors diagnosed in the United States in 2011–2015. Neuro-Oncology. (2018) 20:iv1–iv86. doi: 10.1093/neuonc/noy131 30445539 PMC6129949

[4] Weller M Wick W Aldape K Brada M Berger M Pfister SM. Glioma. Nat Rev Dis Primers. (2015) 1:1–18. doi: 10.1038/nrdp.2015.17 27188790

[5] Honig S Trantakis C Frerich B Sterker I Schober R Meixensberger J. Spheno-orbital meningiomas: outcome after microsurgical treatment: a clinical review of 30 cases. Neurol Res. (2010) 32:314–25. doi: 10.1179/016164109X12464612122614 19726012

[6] Black PM. Benign brain tumors: Meningiomas, pituitary tumors, and acoustic neuromas. Neurol Clinics. (1995) 13:927–52. doi: 10.1016/S0733-8619(18)30026-4 8584005

[7] Fan Y Zhang X Gao C Jiang S Wu H Liu Z. Burden and trends of brain and central nervous system cancer from 1990 to 2019 at the global, regional, and country levels. Arch Public Health. (2022) 80:1–14. doi: 10.1186/s13690-022-00965-5 36115969 PMC9482735

[8] Tahosin MS Sheakh MA Islam T Lima RJ Begum M. Optimizing brain tumor classification through feature selection and hyperparameter tuning in machine learning models. Inf Med Unlocked. (2023) 43:101414. doi: 10.1016/j.imu.2023.101414

[9] Fülöp T Győrfi Á Csaholczi S Kovács L Szilágyi L. (2020). June. Brain tumor segmentation from multi-spectral MRI data using cascaded ensemble learning, in: 2020 IEEE 15th International Conference of System of Systems Engineering (SoSE), . pp. 531–6. IEEE. doi: 10.1109/SoSE50414.2020.9130550

[10] Stadlbauer A Marhold F Oberndorfer S Heinz G Buchfelder M Kinfe TM. Radiophysiomics: brain tumors classification by machine learning and physiological MRI data. Cancers. (2022) 14:2363. doi: 10.3390/cancers14102363 35625967 PMC9139355

[11] Singh RR Vijh S Chaubey D. (2021). An efficient brain tumor detection using modified tree growth algorithm and random forest method, in: 2021 Sixth International Conference on Image Information Processing (ICIIP), , Vol. Vol. 6. pp. 1–6. IEEE. doi: 10.1109/ICIIP53038.2021.9702692

[12] Haq EU Jianjun H Li K Haq HU Zhang T. An MRI-based deep learning approach for efficient classification of brain tumors. J Ambient Intell Humanized Comput. (2021) 14:1–22. doi: 10.1007/s12652-021-03535-9

[13] Alanazi MF Ali MU Hussain SJ Zafar A Mohatram M Irfan M. Brain tumor/mass classification framework using magnetic-resonance-imaging-based isolated and developed transfer deep-learning model. Sensors. (2022) 22:372. doi: 10.3390/s22010372 35009911 PMC8749789

[14] Mahmud MI Mamun M Abdelgawad A. A deep analysis of brain tumor detection from mr images using deep learning networks. Algorithms. (2023) 16:176. doi: 10.3390/a16040176

[15] Ahmad B Sun J You Q Palade V Mao Z. Brain tumor classification using a combination of variational autoencoders and generative adversarial networks. Biomedicines. (2022) 10:223. doi: 10.3390/biomedicines10020223 35203433 PMC8869455

[16] Saeedi S Rezayi S Keshavarz H R. Niakan Kalhori S. MRI-based brain tumor detection using convolutional deep learning methods and chosen machine learning techniques. BMC Med Inf Decision Making. (2023) 23:16. doi: 10.1186/s12911-023-02114-6 PMC9872362 36691030

[17] Asiri AA Aamir M Shaf A Ali T Zeeshan M Irfan M. Block-wise neural network for brain tumor identification in magnetic resonance images. Computers Mater Continua. (2022) 73:5735–53. doi: 10.32604/cmc.2022.031747

[18] Dosovitskiy A Beyer L Kolesnikov A Weissenborn D Zhai X Unterthiner T. An image is worth 16x16 words: Transformers for image recognition at scale. arXiv preprint arXiv:2010.11929. (2020). doi: 10.48550/arXiv.2010.11929

[19] Steiner A Kolesnikov A Zhai X Wightman R Uszkoreit J Beyer L. How to train your vit? data, augmentation, and regularization in vision transformers. arXiv preprint arXiv:2106.10270. (2021). doi: 10.48550/arXiv.2106.10270

[20] Dai Y Gao Y Liu F. Transmed: Transformers advance multi-modal medical image classification. Diagnostics. (2021) 11:1384. doi: 10.3390/diagnostics11081384 34441318 PMC8391808

[21] Gheflati B Rivaz H. (2022). July. Vision transformers for classification of breast ultrasound images, in: 2022 44th Annual International Conference of the IEEE Engineering in Medicine & Biology Society (EMBC), . pp. 480–3. IEEE. doi: 10.48550/arXiv.2110.14731 36086171

[22] Tummala S Kadry S Bukhari SAC Rauf HT. Classification of brain tumor from magnetic resonance imaging using vision transformers ensembling. Curr Oncol. (2022) 29:7498–511. doi: 10.3390/curroncol29100590 PMC9600395 36290867

[23] Asiri AA Shaf A Ali T Pasha MA Aamir M Irfan M. Advancing brain tumor classification through fine-tuned vision transformers: A comparative study of pre-trained models. Sensors. (2023) 23:7913. doi: 10.3390/s23187913 37765970 PMC10535333

[24] Badža MM Barjaktarović MČ. Classification of brain tumors from MRI images using a convolutional neural network. Appl Sci. (2020) 10:1999. doi: 10.3390/app10061999

[25] Ge C Gu IYH Jakola AS Yang J. Enlarged training dataset by pairwise GANs for molecular-based brain tumor classification. IEEE Access. (2020) 8:22560–70. doi: 10.1109/Access.6287639

[26] Huang Z Du X Chen L Li Y Liu M Chou Y. "Convolutional neural network based on complex networks for brain tumor image vlassification with a modified activation function". IEEE (2020) vol. 8, pp. 89281–89290. doi: 10.1109/ACCESS.2020.2993618

[27] Çinarer G Emiroğlu BG Yurttakal AH. Prediction of glioma grades using deep learning with wavelet radiomic features. Appl Sci. (2020) 10:6296. doi: 10.3390/app10186296

[28] Ayadi W Charfi I Elhamzi W Atri M. Brain tumor classification based on hybrid approach. Visual Comput. (2022) 38:107–17. doi: 10.1007/s00371-020-02005-1

[29] Cheng J Huang W Cao S Yang R Yang W Yun Z. Enhanced performance of brain tumor classification via tumor region augmentation and partition. PloS One. (2015) 10:e0140381. doi: 10.1371/journal.pone.0140381 26447861 PMC4598126

[30] Cheng J Yang W Huang M Huang W Jiang J Zhou Y. Retrieval of brain tumors by adaptive spatial pooling and fisher vector representation. PloS One. (2016) 11:e0157112. doi: 10.1371/journal.pone.0157112 27273091 PMC4894628

[31] Cheng J. Brain Tumor Dataset(2017). Available online at: https://figshare.com/articles/dataset/brain_tumor_dataset/1512427.

[32] Bakas S Akbari H Sotiras A Bilello M Rozycki M Kirby JS. Advancing the cancer genome atlas glioma MRI collections with expert segmentation labels and radiomic features. Sci Data. (2017) 4:1–13. doi: 10.1038/sdata.2017.117 PMC5685212 28872634

[33] Clark K Vendt B Smith K Freymann J Kirby J Koppel P. The Cancer Imaging Archive (TCIA): maintaining and operating a public information repository. J Digital Imaging. (2013) 26:1045–57. doi: 10.1007/s10278-013-9622-7 PMC3824915 23884657

