# Supplementary figures and images for: A fine-tuned vision transformer based enhanced multi-class brain tumor classification using MRI scan imagery
Source: Front Oncol. 2024 Jul 18;14:1400341. doi: 10.3389/fonc.2024.1400341 (PMC11291226; doi:10.3389/fonc.2024.1400341)

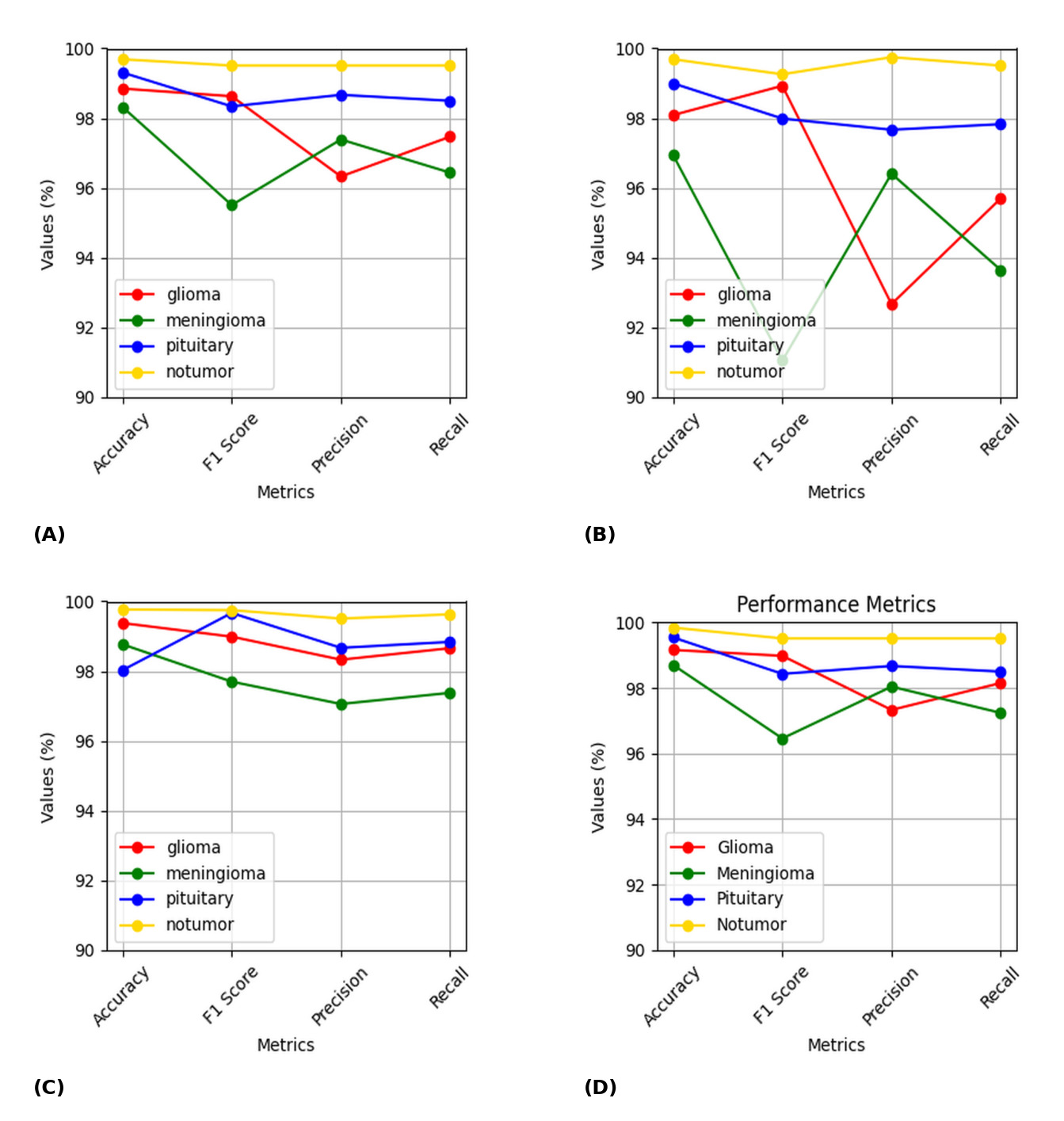

Supplement: Supplementary file 1 [file Image_1.jpeg]

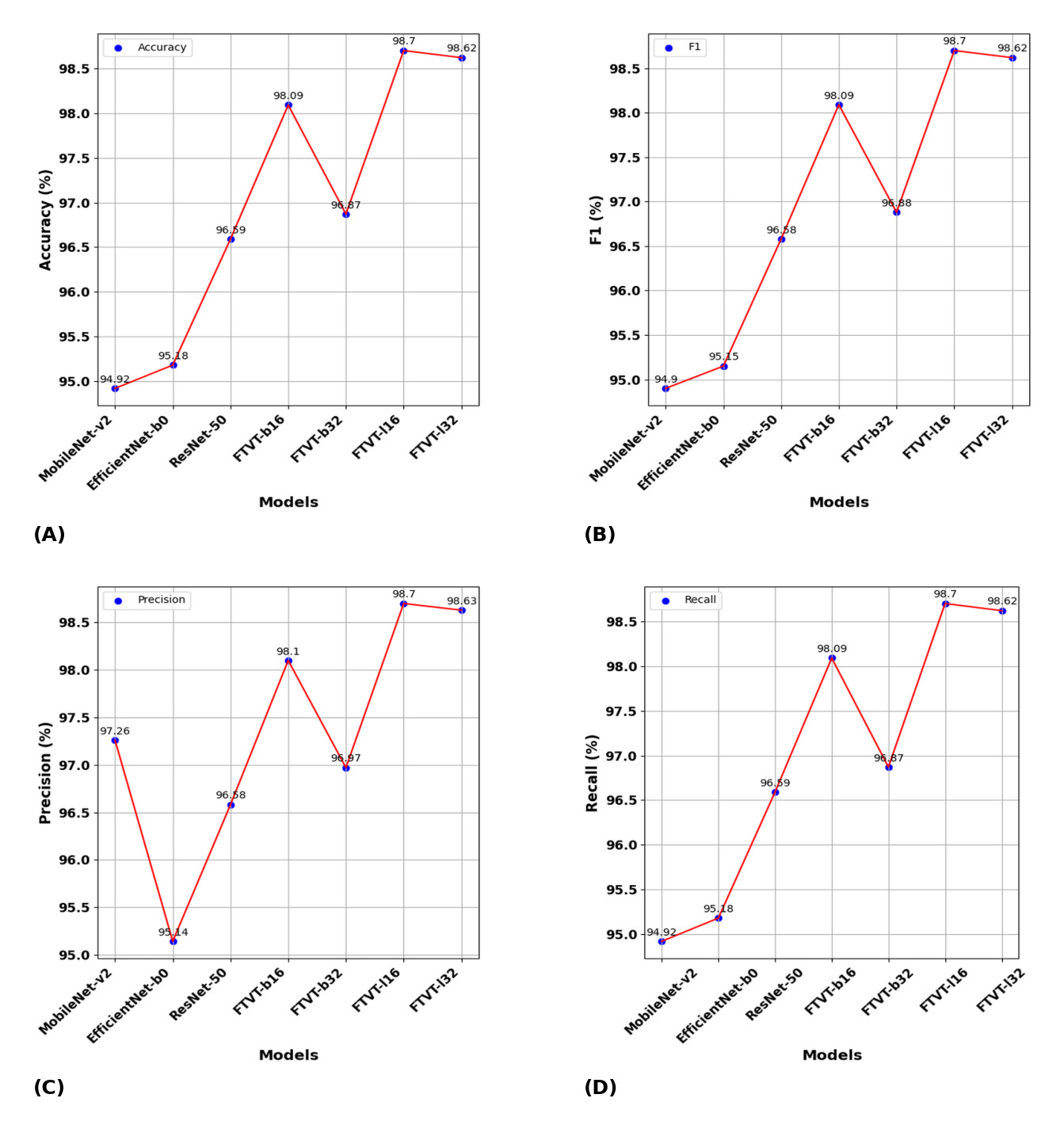

Supplement: Supplementary file 2 [file Image_2.jpeg]

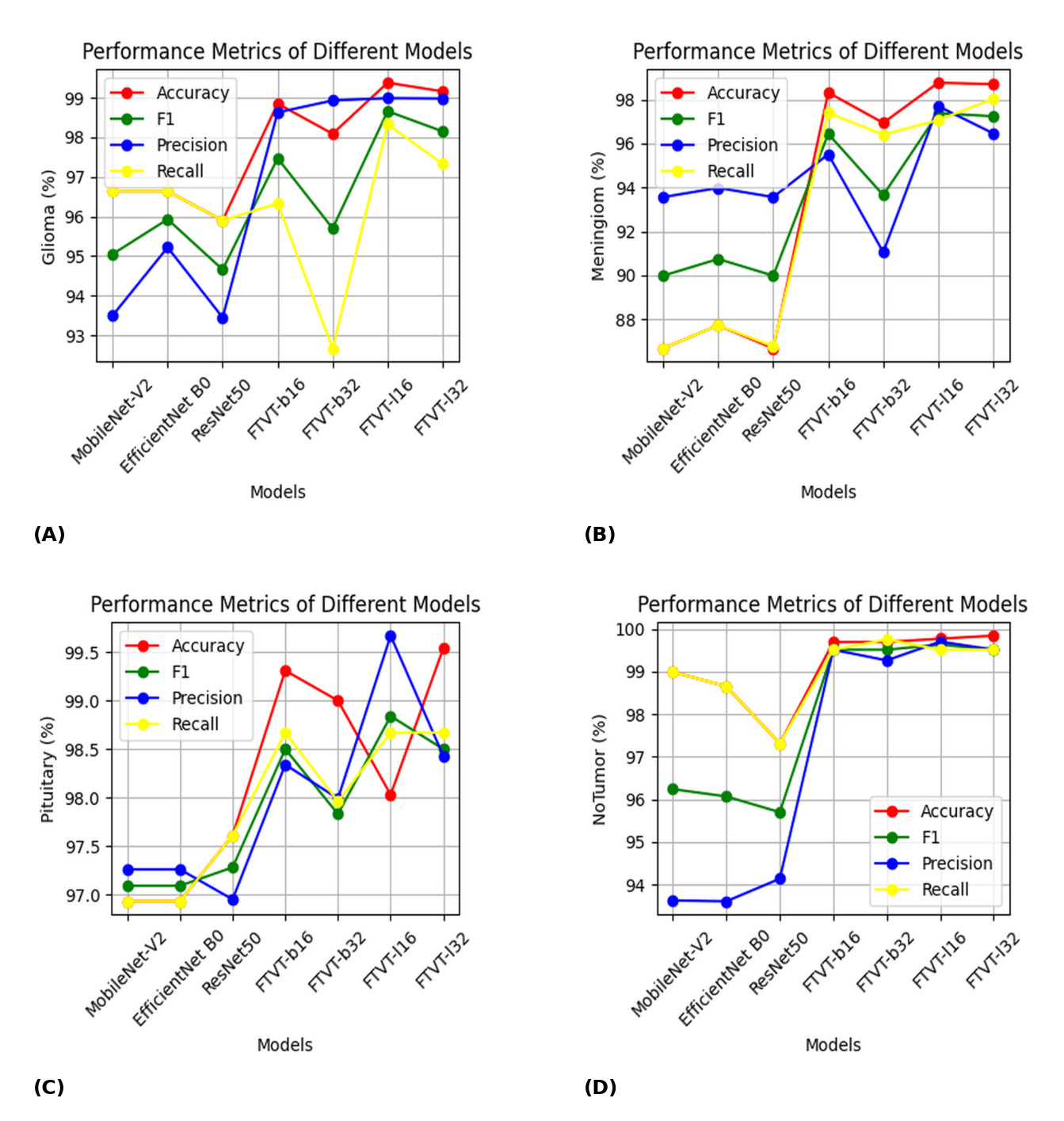

Supplement: Supplementary file 3 [file Image_3.jpeg]
